# Mice Lacking Platelet-Derived Growth Factor D Display a Mild Vascular Phenotype

**DOI:** 10.1371/journal.pone.0152276

**Published:** 2016-03-31

**Authors:** Hanna Gladh, Erika Bergsten Folestad, Lars Muhl, Monika Ehnman, Philip Tannenberg, Anna-Lisa Lawrence, Christer Betsholtz, Ulf Eriksson

**Affiliations:** 1 Department of Medical Biochemistry and Biophysics, Division of Vascular Biology, Karolinska Institutet, Stockholm, Sweden; 2 Department of Surgical Sciences, Division of Vascular Surgery, Karolinska University Hospital, Stockholm, Sweden; 3 Department of Immunology, Genetics and Pathology, Rudbeck Laboratory, Uppsala University, Uppsala, Sweden; Albert Einstein College of Medicine, UNITED STATES

## Abstract

Platelet-derived growth factor D (PDGF-D) is the most recently discovered member of the PDGF family. PDGF-D signals through PDGF receptor β, but its biological role remains largely unknown. In contrast to other members of the PDGF family of growth factors, which have been extensively investigated using different knockout approaches in mice, PDGF-D has until now not been characterized by gene inactivation in mice. Here, we present the phenotype of a constitutive *Pdgfd* knockout mouse model (*Pdgfd*^*-/-*^), carrying a *LacZ* reporter used to visualize *Pdgfd* promoter activity. Inactivation of the *Pdgfd* gene resulted in a mild phenotype in C57BL/6 mice, and the offspring was viable, fertile and generally in good health. We show that *Pdgfd* reporter gene activity was consistently localized to vascular structures in both postnatal and adult tissues. The expression was predominantly arterial, often localizing to vascular bifurcations. Endothelial cells appeared to be the dominating source for *Pdgfd*, but reporter gene activity was occasionally also found in subpopulations of mural cells. Tissue-specific analyses of vascular structures revealed that NG2-expressing pericytes of the cardiac vasculature were disorganized in *Pdgfd*^*-/-*^ mice. Furthermore, *Pdgfd*^*-/-*^ mice also had a slightly elevated blood pressure. In summary, the vascular expression pattern together with morphological changes in NG2-expressing cells, and the increase in blood pressure, support a function for PDGF-D in regulating systemic arterial blood pressure, and suggests a role in maintaining vascular homeostasis.

## Introduction

The platelet-derived growth factor (PDGF) family consists of four ligands, PDGF-A to -D. They are all secreted as disulfide-bonded homo- or heterodimers, PDGF-AA, -AB, -BB, -CC and–DD, and exert their biological functions by binding to and signaling via two tyrosine kinase receptors, PDGF receptor (PDGFR)α and PDGFRβ [1–4]. The biological function of the PDGF family and their receptors has been extensively investigated, with the exception of PDGF-D [5].

PDGF-D shares the conserved growth factor domain with the other members of the family [3, 4]. PDGF-C and PDGF-D also contain an additional N-terminal CUB domain that requires proteolytic removal to enable receptor binding, and are thus secreted as latent dimers [6]. Serine proteases such as urokinase-type plasminogen activator (uPA) and matriptase can activate PDGF-D [7–9]. PDGF-D is the only ligand that binds specifically to, and induces downstream signaling via PDGFRβ [3]. Deletion of the genes encoding the different PDGF ligands and receptors have shown that they are essential during embryonic development. PDGF-A and -C signaling via PDGFRα are broadly required during organogenesis [5]. The PDGF-B and PDGFRβ deficient mice show similar phenotypes, as they die prenatally due to hypoplasia of mural cells (pericytes and vascular smooth muscle cells, vSMCs) leading to vascular defects and other complex cardiovascular malformations [5, 10, 11].

The expression pattern of PDGF-D in normal tissue is not well studied, although in adult human tissue, *PDGFD* mRNA expression has been detected in relatively high levels in adrenal gland, pancreas and heart [3, 4]. In mice, heart, kidney and lung have been reported as high *Pdgfd* expressing organs [12]. Similar to PDGF-B and PDGFRβ, PDGF-D is described to have a vascular expression pattern, with expression in normal human arterial endothelium and arterial vSMCs [13, 14]. PDGF-D expression has also been reported in certain epithelial cells [15, 16].

Evidence is pointing to an important function of PDGF-D signaling through PDGFRβ in the vasculature, where PDGF-D emerges as a vascular modulator during pathological conditions. In a rat model of chronic rejection, adeno-associated virus mediated transfer of PDGF-D, but not PDGF-B, resulted in accelerated cardiac allograft vasculopathy and fibrosis [17]. Heart-specific overexpression of PDGF-D caused vascular remodeling with dilated microvessels and proliferation of vSMCs, resulting in cardiac fibrosis, dilated cardiomyopathy and cardiac failure, whereas overexpression of PDGF-B yielded a much milder phenotype [12]. PDGF-D is also capable of stabilizing newly formed vessels through effects on vSMCs and it contributes to neointimal hyperplasia [13, 18]. Furthermore, in several genome-wide association studies, PDGF-D has been associated with coronary artery disease [19–21].

Here we investigate the *Pdgfd* expression pattern and effects of the PDGF-D deficiency in a new constitutive *Pdgfd* knockout (*Pdgfd*^*-/-*^*)* mouse line, containing a *LacZ* reporter gene allowing visualization of *Pdgfd* expression sites. We show that PDGF-D deficient mice are viable, and thus offer the opportunity to study the physiological and pathological roles of PDGF-D/PDGFRβ signaling in adult mice. Moreover, we demonstrate a vascular, predominantly arterial, expression pattern of *Pdgfd* in all investigated organs. Furthermore, we show morphological changes in NG2-expressing cells within cardiac tissue and demonstrate an increase in blood pressure in *Pdgfd* deficient mice. Taken together, these findings suggest a regulatory function for PDGF-D in the systemic arterial blood pressure, and furthermore suggest a role in maintenance of vascular homeostasis.

## Materials and Methods

### Ethics statement

This study was carried out in accordance with the Swedish legislation of animal welfare. The protocols for this study were approved by the Stockholm North Ethical Committee on Animal Research (ethical permit numbers N187/13, N140/13, N451/12, N127/11, N128/11, N325/11, N140/10, N188/10 and N231/09). All animals used in this study were monitored daily by the animal facility staff. Whenever illness was seen, or suspected, animals were assessed according to the Karolinska Institutet extended assessment checklist for small rodents and rabbits, and euthanized if a total score of more than 0.3 was reached [22, 23]. Animals were kept in a pathogen free animal facility with controlled temperature and 12h/12h day-night cycle lightning, communally housed in standard cages with enrichment in form of nesting material and had free access to food/water. All invasive procedures were terminal, and performed under deep anesthesia, or after euthanization. For blood collection using heart puncture, animals were sedated by isoflurane gas. For all other experiments involving organ collection, animals were euthanized by intraperitoneal injection of Hypnorm (2,5 mg/ml Fluanisone, 0,07875 mg/ml Fentanyl citrate) Midazolam (1,25 mg/ml), in dH_2_O, 10 ml/kg. Animals that were not assigned to experiments involving necropsy were euthanized by CO_2_ followed by cervical dislocation.

### Generation, validation and maintenance of *Pdgfd* null mice

The *Pdgfd*^*+/-*^ mouse strain was generated by Regeneron Pharmaceuticals Inc, using the VelociGene technology [24]. In the *Pdgfd* gene 0.1 kb from the ATG start codon to the 3’-end of exon1 was replaced by a *LacZ* reporter gene cassette generated using a BAC-based targeting vector containing a *Neomycin* resistance cassette (*Ub1 Em7 Neo cassette*) flanked by two *LoxP* sites. Successfully targeted 129S6SvEv/C57BL6F1 ES clones were selected based on neomycin resistance, and screened by a “loss-of-native-allele” (LOA) assay described in [24], using real-time quantitative PCR (qPCR). A *Pdgfd*-targeted positive clone (5120C-F10) with a LOA score of 0.92 was injected into mouse blastocysts to obtain chimeric mice. F1 heterozygotes males and females were shipped to Karolinska Institutet and backcrossed for >10 generations with C57BL/6J (Jackson Laboratories) to establish a knockout mouse line, in which the *Neomycin* resistance cassette was not removed. Genotyping was done by conventional PCR of genomic DNA from ear, or tail biopsies to detect the *Pdgfd* wildtype allele (generating a 289-bp fragment), or the *Pdgfd* knockout allele (generating a 387-bp fragment), respectively. A forward primer, located upstream (-252 bp) of the ATG start codon within exon 1 of *Pdgfd*, was used to detect both alleles. The genomic reverse primer to detect the wildtype allele is located 15 bp downstream of the ATG-codon and the cassette primer to detect the inserted allele is located 111 bp downstream of the ATG within the cassette. Absence of *Pdgfd* exon1 mRNA in *Pdgfd*^-/-^ mouse hearts was confirmed by quantitative real time-PCR (qPCR) (n = 3 per genotype). All primer sequences are found in S1 Table.

The mouse strain was maintained by heterozygous breeding and continuous backcrossing with C57BL/6J mice. A cohort of animals from heterozygous breeding was kept up to the maximum age of 18 months according to our ethical permit. For rapid expansion, offspring from different heterozygous breeding were set up as ≥5 homozygous breeding pairs per genotype (*Pdgfd*^*+/+*^ x *Pdgfd*^*+/+*^; *Pdgfd*^*-/-*^ x *Pdgfd*^*-/-*^*)*. First generation animals were used for experiments, but not for further breeding. Pups were counted at birth and surviving animals were then weighed weekly, or once every second weeks for a period of up to 16 weeks. After the 16 weeks monitoring period, animals were assigned to other experimental procedures in order to reduce the number of animals used.

### Organ and serum collection

For RNA extraction, organs were snap frozen and further processed as described in the next section (see below). For histology, animals were perfused with dPBS or HBSS (Gibco) followed by 0,2% paraformaldehyde (PFA) in dPBS (for cryosectioning), or in 4% PFA in dPBS (for paraffin embedding) prior to organ collection. Before paraffin embedding, organs were postfixed in 4% PFA for 24 h.

### RNA isolation and qPCR

Snap frozen organs were homogenized in TRIzol® Reagent (Ambion). After addition of 0.2 volumes of chloroform, RNA was extracted using the RNeasy Mini Kit (Qiagen), according to manufacturer’s instructions. Genomic DNA was removed using the RNAse-free DNase kit (Qiagen), and cDNA was generated using the iScript™ cDNA Synthesis Kit (Bio-Rad) using a VWR UnoCycler Thermal Cycler. Final RNA concentrations were measured using a Nanodrop ND-1000. qPCR was performed using KAPA SYBR® FAST Universal 2X qPCR Master Mix (Kapa Biosystems), on a Rotor-Gene Q real-time cycler (Qiagen). L19 was used as reference gene, and the ΔΔCT method was used to calculate relative expression. *Pdgfd* expression analysis was performed in heterozygotes (n≥3 except for bone, cerebrum, eye, kidney, liver, soleus, quadriceps, skin, spleen, thymus and trachea, n = 2, and uterus, n = 1). The expression analysis of *Pecam1*, *Cspg4*, *Desmin*, *Notch1* and *Gata4* in heart was based on three animals per genotype. The primer sequences are presented in S1 Table.

### X-gal staining

X-gal staining was performed on whole mounts (postnatal day 4 or 3 months old heterozygous animals), or 12–16 μm thick sections (3–6 months old heterozygous animals). Tissues were postfixed in 1–2% PFA in PBS, or in 0.2–0.5% glutaraldehyde PBS (for sections, 10 min; for whole mounts, 20 min) at +4°C and permeabilized with 2 mM MgCl_2_, 0.02% Nonidet P40 substitute, 0.01% deoxycholic acid sodium salt in PBS. Incubations with the X-gal reagent (5-bromo-4-chloro-3-indolyl-β-D-galactopyranoside, Promega) was performed o/n at +37°C. Sections were then either subjected to further staining according to standard protocols below, or mounted.

### Immunohistochemistry

For immunohistochemical (IHC) staining of cryosections, antigen retrieval was performed using 0.05% trypsin-EDTA (Gibco, Life Technologies), for 20 min at +37°C. Endogenous peroxidases were quenched by incubation in 0.3% H_2_O_2_ in TNT buffer (0.05% Tween 20, 0.1 M Tris pH 7.5, 0.15 M NaCl), for 30 min. Sections were incubated with primary antibody (+4°C, o/n), and secondary antibody (RT, for 30 min), using the TSA indirect kit (NEN Life Science), according to the manufacturers instructions. The signal was developed using 3,3'-diaminobenzidine (DAB Vector Laboratories, Inc.). Sections were either directly dehydrated and mounted with X-tra-kit mounting medium (Medite), or further counterstained with hematoxylin and/or eosin (H&E) according to standard protocol, followed by bleaching by brief dipping into 36 mM HCl in 70% EtOH.

For detection of PDGF-D expression, an affinity-purified polyclonal antibody (anti-PDGF-D rabbit 626) made in-house was used. The antibody was generated by injecting human full-length PDGF-D into a rabbit and affinity-purified as described in [2, 3]. Paraffin sections were rehydrated and subjected to heat induced antigen retrieval in citrate buffer pH 6.1, (Target retrieval solution S1699, *DAKO*) for 60 min in steamer prior to the trypsin step, quenching of peroxidases and antibody incubations as described for cryosections. The antibody signal was amplified using the Tyramide Signal Amplification (TSA) kit (*PerkinElmer*) according to manufacturers instructions. The signal was developed and sections were mounted as described above.

### Immunofluorescence stainings

Cryosections (10–16 μm) or vibratome sections (50 μm) sections were permeabilized in blocking buffer (1% BSA, 0.5% Triton X-100 in PBS) prior to incubation with primary antibody (o/n, +4°C), and fluorescent-labeled secondary antibody (1h, RT). Paraffin sections (4.5 μm) were permeabilized after antigen retrieval with 0.025% trypsin-EDTA (Gibco, Life Technologies), for 5 min at 59°C. For insulin/glucagon staining, an additional retrieval step was performed by heating in a citrate buffer pH 6 (Target retrieval solution S2369, Dako) in a microwave oven for 2x5 min.

Primary antibodies used are listed in S2 Table. For immunofluorescent detection of primary antibodies made in rat, a secondary antibody anti-rat 594 (R&D, 1:400) was used. For all other detections, appropriate secondary antibodies were chosen from the donkey Alexa Fluor® -488, -594 and -647-conjugated antibodies (ThermoFisher Scientific), diluted 1:1000 (cryosections), or 1:200–1:400 (paraffin). Cell nuclei were visualized with DAPI (1:1000) and mounting was performed using ProLong Gold Antifade Reagent (Molecular Probes).

### Intravenous injection and visualization of fluorescent tracer

Alexa Fluor® 555-conjugated Cadaverine (Invitrogen) was injected intravenously into the tail vein of adult mice using 0.2 mg/animal in 100 μl dPBS (Gibco), (3 months, n = 4 per genotype), and allowed to circulate for 2–4 h. For *in situ* detection of tracer, heart and kidney were postfixed in 4% PFA at +4 °C for 4 h. 50 μm thick vibratome sections were immunostained with an anti-podocalyxin antibody as described above. To verify successful systemic distribution of injected tracer, kidney was used as positive control.

### Imaging of stained tissues

Regular brightfield and immunofluorescence imaging was performed using an inverted AxioObserver Z.1 microscope and equipped with an Apotome unit (Zeiss). The latter was used for immunofluorescence-stained paraffin sections. The X-gal/podocalyxin stainings were imaged with fluorescent and transmitted light simultaneously. Imaging of X-gal/ASMA, PECAM/PDGFRβ, PECAM/NG2 and cadaverine/podocalyxin was performed using an LSM700 (Zeiss) confocal microscope. The blue X-gal precipitate in co-staining with ASMA was captured (as black) using transmitted light. Whole mounts were imaged using a Lumar.V12 with an AxioCam HRc (Zeiss). The software used for acquisition was AxioVision, Zen Black and Zen Blue (Zeiss). Representative images are shown in the figures.

### Image quantification

Quantifications of stainings were performed in AxioVision (Zeiss) or ImageJ. Vascular density was calculated as endothelial (podocalyxin) staining (pixels)/frame area (pixels), while vSMC/pericyte (NG2) staining (pixels) was normalized to endothelial staining (pixels).

Presented data is obtained from tissues stained for PDGFRβ/PECAM (heart), NG2/PECAM (heart) and NG2/podocalyxin (exocrine and endocrine pancreas) and based on at least three animals per genotype and a total frame number of 20–146. Representative images of podocalyxin-stained endocrine pancreas are found in S3 Fig. Pancreatic islet morphology was analyzed on two sections, at least 200 μm apart, from each animal (n = 4 per genotype). All islets were imaged with a 40x objective and encircled to obtain the total islet area. Whole section area was obtained by mosaic imaging at 5x followed by area measurement. Total insulin per section area, insulin area per islet area and insulin/glucagon pixel ratios were calculated from the parameters above.

### Blood pressure measurements

Blood pressure measurements were performed non-invasively using a two-channel CODA Tail cuff High Throughput system (Kent Scientific), as described by the manufacturer. Mice were allowed one week of acclimatization to the equipment to reduce stress. Mice were placed in a restrainer on a heating pad, on day 1–2 for 8–10 min, day 3 for 8–10 min with cuffs on tail, and day 4–5 for 5 min with cuffs on tail followed by 10 measurement cycles (30 s per cycle with 15 s rest in between). On the day of the experiment, the mice were acclimatized 5–10 min, with the equipment in place, prior the measurements. Twenty blood pressure measurement cycles were performed on each animal at the same time each day, for five consecutive days. The first 5 recordings, and any recording showing a tail blood flow below 20 or where the animal had moved during the measurement, were excluded. The experiments were repeated three times with the same magnitude of increase between the groups (n = 4–6 male animals per group, total n = 15 per genotype).

### Dry and wet heart weight

Hearts were weighed upon harvest, then dried for 10 days at 56°C and weighed again, using an analytical scale (Sartorius). *Pdgfd*^*+/+*^ n = 15 and *Pdgfd*^-/-^ n = 16.

### Glucose and insulin tolerance tests

Glucose and insulin tolerance tests were performed on age-matched males (20±3 weeks of age). Glucose (2 mg/g BW in 200 μl) was administered to animals that were fasted o/n (12 h), and insulin (0.75 mU/g BW in 100 μl), was administered to non-fasted animals, by intraperitoneal injection, as previously described [25] (n ≥12 animals per genotype).

### Clinical chemistry

Clinical chemistry was performed by the University Animal Hospital, Swedish University of Agricultural Sciences, Uppsala, Sweden. In mouse serum (n = 5 per sex and genotype), ASAT, ALAT, ALP, albumin and calcium were analyzed with spectrophotometry and the electrolytes (chloride, sodium and potassium) with an ion specific electrode on an automatic multi-analyzer Architect 4000c (Abbott Laboratories), all with commercial reagents (Abbott Laboratories).

### Statistics

The Mendelian ratio was calculated from 350 pups born from heterozygous breeding pairs and statistics were calculated by chi-square test. All other statistics were calculated by Students t-test (for p≤0.05, data was considered significant, * p≤0.05, ** p≤0.01, *** p≤0.001), using either Microsoft Excel or Prism GraphPad.

## Results

### *Pdgfd*^*-/-*^ mice are viable and fertile

**Fig 1:**
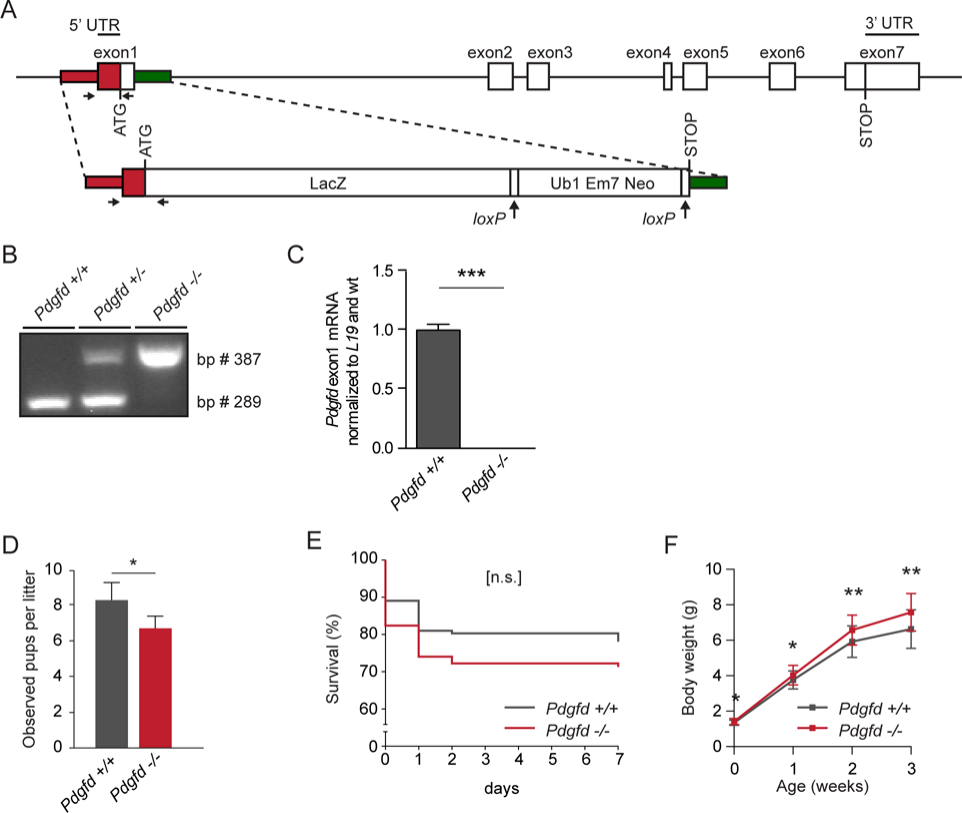
Generation, genotyping and general phenotyping of *Pdgfd*-deficient mice. (A) Schematic outline of the *Pdgfd* gene with exon numbers indicated. The target construct was inserted into exon1. The locations for genotyping primer binding sites are indicated by left and right arrows below exon1 and construct. (B) Genotyping PCR showing the presence of the three possible genotypes, *Pdgfd*^+/+^, *Pdgfd*^+/-^ and *Pdgfd*^-/-^. The wildtype band is 289 bp, and the knockout band is 387 bp. (C) RT-PCR on heart tissue from *Pdgfd*^+/+^ and *Pdgfd*^-/-^ mice showing the lack of exon1 sequences in *Pdgfd* mRNA in *Pdgfd*^-/-^ animals. *L19* was used as the reference gene (*Pdgfd*^*+/+*^ n = 3 and *Pdgfd*^-/-^ n = 3). (D-F) The growth and development of wildtype *and Pdgfd*^*-/-*^ animals from homozygote breedings were followed from birth until 16 weeks of age. (D) Observed pups born per litter, from ≥20 litters per genotype. (E) Survival % of pups born during the first week (*Pdgfd*^*+/+*^ n = 137 and *Pdgfd*^-/-^ n = 108). (F) Weight curves of surviving offspring. Weight of surviving pups was measured every week (0–3 weeks) (*Pdgfd*^*+/+*^ n≥90 and *Pdgfd*^-/-^ n≥80). Error bars indicating standard deviation in (C-D, F).

To investigate the biological function of PDGF-D, a constitutive *Pdgfd* knockout mouse strain was generated by replacing exon1 in the *Pdgfd* gene with a *LacZ* expression cassette (Fig 1A). Genotypes were confirmed using PCR of genomic DNA (Fig 1B). The lack of *Pdgfd* exon1 mRNA expression in *Pdgfd*^-/-^ tissue from heart was verified by qPCR (Fig 1C). In heterozygous breedings of mice on C57BL/6 background, the offspring (*Pdgfd*^+/+^, *Pdgfd*^+/-^, and *Pdgfd*^*-/-*^) were born in the expected Mendelian ratio (S3 Table), and the mice displayed an overall good general health (followed up to 18 months of age). The finding that lack of *Pdgfd* expression does not generate a severe or lethal phenotype showed that PDGF-D signaling is not essential during embryonic development. To study possible phenotype(s) in the PDGF-D deficient mice, first generation homozygous mice derived from the heterozygous main breedings were set up as new breeding pairs and proved to be fertile. Growth and development of the offspring were studied for 16 weeks, and no gross abnormalities were observed during the study period. However, *Pdgfd*^-/-^ mice were born at a lower average number per litter, compared with wildtype control litters (Fig 1D), and the *Pdgfd*^-/-^ mice showed a trend towards reduced survival during the first 7 days (Fig 1E). Furthermore, surviving *Pdgfd*^-/-^ animals were significantly heavier than wildtype controls during the first 3 weeks (Fig 1F), and throughout the 16-week period of observation (S1 Fig). The lack of an obvious phenotype in adult mice indicated that PDGF-D is not of vital importance for postnatal life.

### *Pdgfd* promoter-driven *LacZ* expression displays a consistent vascular localization

**Fig 2:**
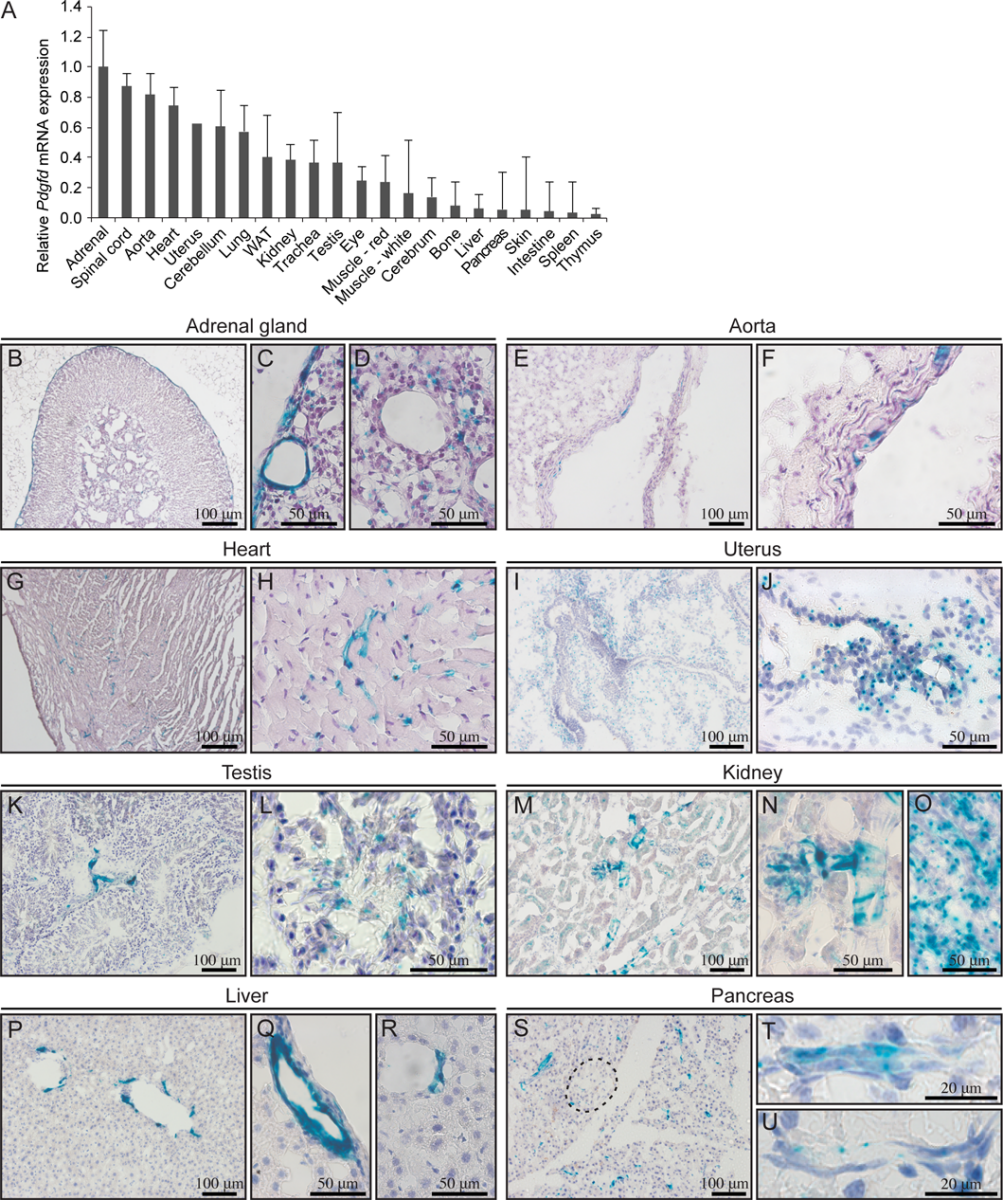
*Pdgfd* promoter-driven *LacZ* expression displays a consistent vascular localization pattern. (A) qPCR analysis on RNA from different tissues from 13 weeks old adult wild-type C57BL/6 mice. Total expression of *Pdgfd* mRNA measured as % of *L19* and normalized to adrenal gland (peak organ). (B-U) X-gal staining (blue) of frozen sections from different tissues from 21 weeks old *Pdgfd*^+/-^ mice, counterstained with hematoxylin. (B) Adrenal gland. (C) Adrenal cortex. (D) Adrenal medulla. (E) Aorta. (F) Aorta, higher magnification. (G) Heart. (H) Heart, myocardium, higher magnification. (I) Uterus. (J) Uterus, endometrium. (K) Testis. (L) Testis, seminiferous tubules. (M) Kidney. (N) Kidney, glomerulus and afferent arteriole. (O) Renal papilla. (P) Liver. (Q) Liver, bile duct. (R) Liver, blood vessel. (S) Pancreas, endocrine islet indicated by the dashed line. (T) Pancreatic duct (U) Pancreas, blood vessel. Scale bars 20, 50 or 100 μm. Error bars in (A) indicate standard deviation.

To identify sites of interest for studying PDGF-D function, a general expression analysis was performed. *Pdgfd* mRNA from a variety of adult tissues was analyzed by qPCR (Fig 2A). The result showed that *Pdgfd* mRNA was most abundantly expressed in adrenal gland, spinal cord, aorta, heart, uterus, cerebellum and lung. Moderate expression was found in white adipose tissue (WAT), kidney, trachea, testis, eye, red and white muscle and liver, while lower expression was observed in cerebrum, bone, liver, pancreas, skin, intestine, spleen and thymus.

To further determine the expression pattern of *Pdgfd*, a comprehensive histological analysis was performed. The *Pdgfd* knockout construct contains the *LacZ* reporter gene encoding β-galactosidase that enables detection of *Pdgfd* expression. Through its enzymatic activity the substrate X-gal yields a blue insoluble product in the tissue sections. From here on, when discussing X-gal staining, *Pdgfd* promoter driven expression of the *LacZ* reporter gene in heterozygous animals will be denoted as *LacZ* expression.

Enzymatic staining for *LacZ* expression on frozen sections from *Pdgfd*^+/-^ tissues (Fig 2B–2U) showed expression in adrenal gland, aorta, heart, uterus, testis, kidney, liver and pancreas. Corresponding sections from wildtype mice were negative for X-gal staining. The adrenal gland (Fig 2B) showed *LacZ* expression in the fibrous capsule (Fig 2C), as well as in larger blood vessels in the outer adrenal cortex (Fig 2C) and in smaller blood vessels within the medulla (Fig 2D). In the aorta, scattered X-gal staining was observed in the different layers of the aorta (Fig 2E and 2F), with stronger staining found in cells close to the lumen and sparser staining in the outer layers (Fig 2F). In the heart, *LacZ* expression was generally seen in blood vessels of the myocardium (Fig 2G) and appeared stronger in some, but not all blood vessel bifurcations (Fig 2H). In the uterus, *LacZ* expression was found mainly in the endometrium (Fig 2I and 2J) although occasional vascular staining was observed. The testis showed expression of *LacZ* in vascular bifurcations (Fig 2K) and in seminiferous tubules containing spermatids of a more mature state (Fig 2L). In kidney, *LacZ* expression was observed in the vasculature including the glomerular afferent arteriole, in glomeruli and in certain tubuli (Fig 2M). At higher magnification, *LacZ* expression revealed a distinct banding pattern in the afferent arterioles, and in the glomerulus, staining appeared to be located in the mesangial cells (Fig 2N). Intense *LacZ* expression was also observed in the renal papilla (Fig 2O). In the liver (Fig 2P), strong *LacZ* expression was found in bile ducts (Fig 2Q) and weaker vascular *LacZ* expression was observed (Fig 2R). In the pancreas (Fig 2S), *LacZ* expression appeared strong in pancreatic ducts (Fig 2T), and somewhat weaker in blood vessels (Fig 2U). These findings and further description of *LacZ* expression in additional tissues are summarized in Table 1. Furthermore immunohistochemical stainings of wildtype heart, kidney and pancreas, using a polyclonal anti-PDGF-D antibody verified protein expression in most, but not all, structures identified by X-gal staining, while *Pdgfd*^*-/-*^ tissues showed no, or only limited, staining at the corresponding sites (S2 Fig). In *Pdgfd*^*+/+*^ heart, PDGF-D expression was detected in coronary arteries and branches (S2A and S2B Fig, arrows) as well as in a pattern corresponding to the capillary X-gal staining seen in the myocardium (S2E and S2F Fig, arrows). In *Pdgfd*^*-/-*^ heart tissue weak staining was seen in coronary arteries (S2C and S2D Fig, arrows) while staining in the myocardium was absent (S2G and S2H Fig). In wildtype pancreas, PDGF-D staining was seen in the capillaries of the exocrine pancreas (S2I and S2J Fig, arrows) and in kidney expression was found in certain tubuli (S2M and S2N Fig, arrows). PDGF-D stained tissue from *Pdgfd*^*-/-*^ pancreas or kidney showed minor background staining (S2K and S2L and S2O and S2P Fig). The reason that visualization of *Pdgfd* expression using the LacZ reporter showed a wider expression pattern than the antibody stainings is probably a reflection of the higher sensitivity of the reporter technique, or that the PDGF-D protein has a short half life in the tissues.

**Table 1:** *Pdgfd*^*LacZ*^ expression in selected organs.

Pdgfd^+/LacZ^ expression pattern (adult)	Expression level (0 - ++++)
Adrenal*	Capsule ++
	Cortex—vascular +
	Medulla ++
Aorta	Tunica media +++
	Endothelium +
Cerebellum	Purkinje cell layer +++
	Molecular layer +
Cerebrum	Vascular +
	Raphe nucleus/3:rd ventricle wall +++
	Area postrema/4:th ventricle wall +
	Hippocampus +
Eye	Vascular (arterial) ++
	Optic nerve ++
Heart	Atria—vascular +
	Myocardium—vascular +++
	Myocardium—arterial +++
	Myocardium—venous +
Kidney*	Cortex—certain tubuli +
	Glomerular afferent arteriole +++
	Glomerulus ++
	Renal papilla—epithelium ++++
	Vascular ++
Liver*	Vascular +
	Bile ducts—epithelium ++++
Muscle—Quadriceps	Vascular +
Muscle—Soleus	Vascular +
	Vascular +++
Pancreas*	Exocrine ++
	Endocrine +
	Vascular ++
	Intercalated and interlobular ducts—epithelium ++
Pituitary gland	++++
Intestine	Vascular +
	Villi—central capillary +
	Villi—occasional cells +++
Spinal cord	Vascular—non-capillary +
Spleen	Vascular—non-capillary +
Testis*	Tubuli/spermatids, stage dependent (0)-(+++)
	Vascular ++
Uterus*	Endometrium +++
	Vascular +
Ureter	Basal layer +++
	Epithelium +
White adipose tissue (WAT)	Vascular +

*Pdgfd*^*LacZ*^ staining, ranging from undetectable (0) to abundantly expressed (++++).

* Image in (Fig 2)

In summary, several interesting *Pdgfd* expression patterns were found, such as strong staining in the vascular system of all tissues investigated, and in various epithelial tissues. The staining intensity varied in the different vascular beds, but the common expression in the blood vessels suggested that PDGF-D might have an important function in the vasculature. A more detailed expression analysis and physiological experiments were therefore performed with special emphasis on the vasculature.

### *Pdgfd* is highly expressed in cardiac vasculature, particularly in arterial bifurcations

The general expression analysis showed that the heart is an organ with high expression of *Pdgfd*, which is supported by previously published data [3, 4, 12].

**Fig 3:**
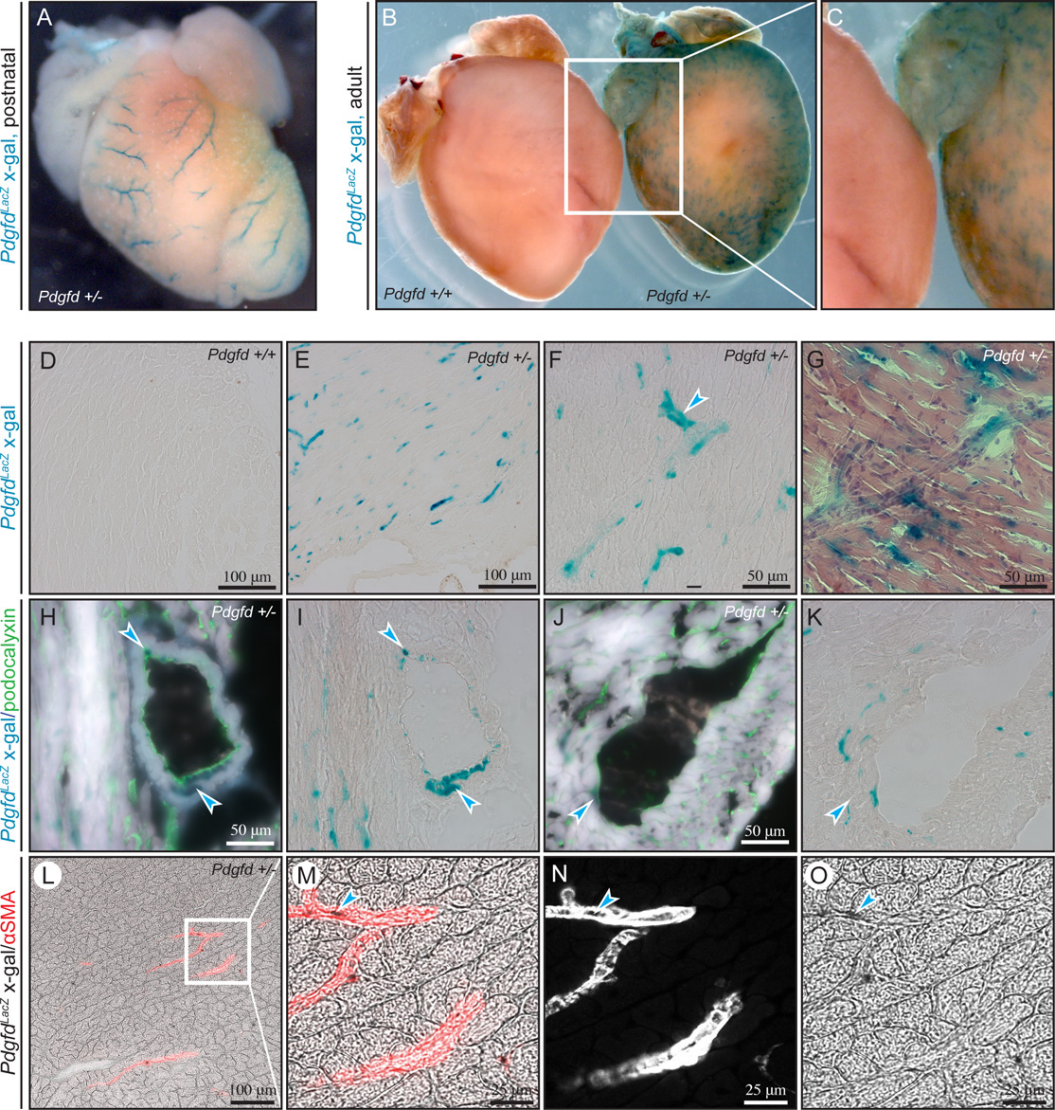
*Pdgfd* is highly expressed in the cardiac vasculature, particularly in arterial bifurcations. Analysis of heart, whole mounts and sections from wildtype and *Pdgfd*^+/-^ mice. (A) Postnatal (day P4) whole mount heart showing X-gal staining (blue) (B) Adult whole mount hearts from 16 weeks old mice *Pdgfd*^+/+^ and *Pdgfd*^+/-^ mice showing X-gal staining. (C) Magnification from (B). (D-G) Representative images of sections from heart showing X-gal staining. (D) *Pdgfd*^+/+^, control. (E) Overview of *Pdgfd*^+/-^ heart. (F) Higher magnification of *Pdgfd*^+/-^ heart, strong staining in blood vessel bifurcation (arrow). (G) *Pdgfd*^+/-^ counterstained with hematoxylin and eosin. (H-K) Representative images of *Pdgfd*^+/-^ heart sections from 21 weeks old mice, showing podocalyxin (H, J) staining as a marker of endothelial cells (green) and X-gal staining (blue). (H) Artery showing X-gal co-staining with podocalyxin (upper arrow) and X-gal staining outside of podocalyxin staining (lower arrow) and (I) in the same section, artery showing X-gal without podocalyxin. (J) A vein showing limited X-gal staining (arrow), and (K) the same section, without podocalyxin staining. (L-O) Representative confocal images of a *Pdgfd*^*+/-*^ heart section from 16 weeks old mice showing immunofluorescent alpha-smooth muscle actin (αSMA) and enzymatic X-gal staining visualized by transmitted light. (L) Arteries showing αSMA (red) and X-gal staining (black). (M-O) Magnifications from (L), arrows pointing at black X-gal staining. (M) Arteries showing αSMA (red) and X-gal staining (black). (N) αSMA (white), no transmitted light. (O) X-gal staining (black). Scale bars 100 μm.

To complement these observations, *Pdgfd* driven *LacZ* expression was investigated in whole mount hearts from postnatal (P4) and adult animals (Fig 3A–3C). Both postnatal and adult hearts showed distinct and strong vascular *LacZ* expression. In postnatal heart, *LacZ* expression was undetectable in the atrium, while distinct staining was observed in epicardial coronary vessels (Fig 3A). As the heart developed from postnatal to adult, *LacZ* expression became more widespread throughout the heart, including the atria. LacZ expression in the epicardial coronary vessels became less prominent while smaller vessels displayed a more pronounced *LacZ* expression (Fig 3B and 3C). Wildtype littermate controls were negative for *LacZ* expression (Fig 3B–3D).

A more detailed analysis of heart sections stained for *LacZ* expression showed a widespread expression in the myocardial vasculature (Fig 3E). At higher magnification, the *LacZ* expression appeared to be strong in medium-sized blood vessels, likely arterioles. Interestingly, the *LacZ* expression was particularly prominent at vascular bifurcations, especially in the smaller vessel just after the branching point (Fig 3F, arrow). To provide more details of the tissue localization of the staining, X-gal stained sections were counterstained with hematoxylin and eosin. By morphology, the vascular *LacZ* expression appeared strong in arterioles, particularly at branching points, and weaker as the vessels became smaller (Fig 3G).

In order to classify the blood vessels that express *Pdgfd* and describe its cellular expression more closely, X-gal stained heart sections were subsequently stained for podocalyxin expression to identify endothelial cells. Intense *LacZ* expression was observed in arteries (Fig 3H and 3I), while little expression was found in veins (Fig 3J and 3K, arrows). Furthermore, the expression co-localized with podocalyxin at some sites (Fig 3H, upper arrow), while at branching points strong focal expression was clearly located outside of the podocalyxin staining, likely in the vSMCs surrounding the vessel (Fig 3H, lower arrow).

Confocal images from sections stained for *LacZ* expression and immunofluorescence-labeled αSMA to visualize vSMCs, verified that predominantly arteries were stained (Fig 3L–3O). Moreover, in smaller αSMA-expressing arterioles, the *LacZ* expression was often found within the αSMA staining (3M-O, arrows), indicating that endothelial cells express *Pdgfd* at these sites.

In summary, *Pdgfd* was abundantly expressed in the heart vasculature, and the expression localized mainly to arteriolar vessels. Moreover, both endothelial cells and perivascular cells, such as vSMCs were found to express *Pdgfd*.

### *Pdgfd* is predominantly expressed in arterial endothelial cells

**Fig 4:**
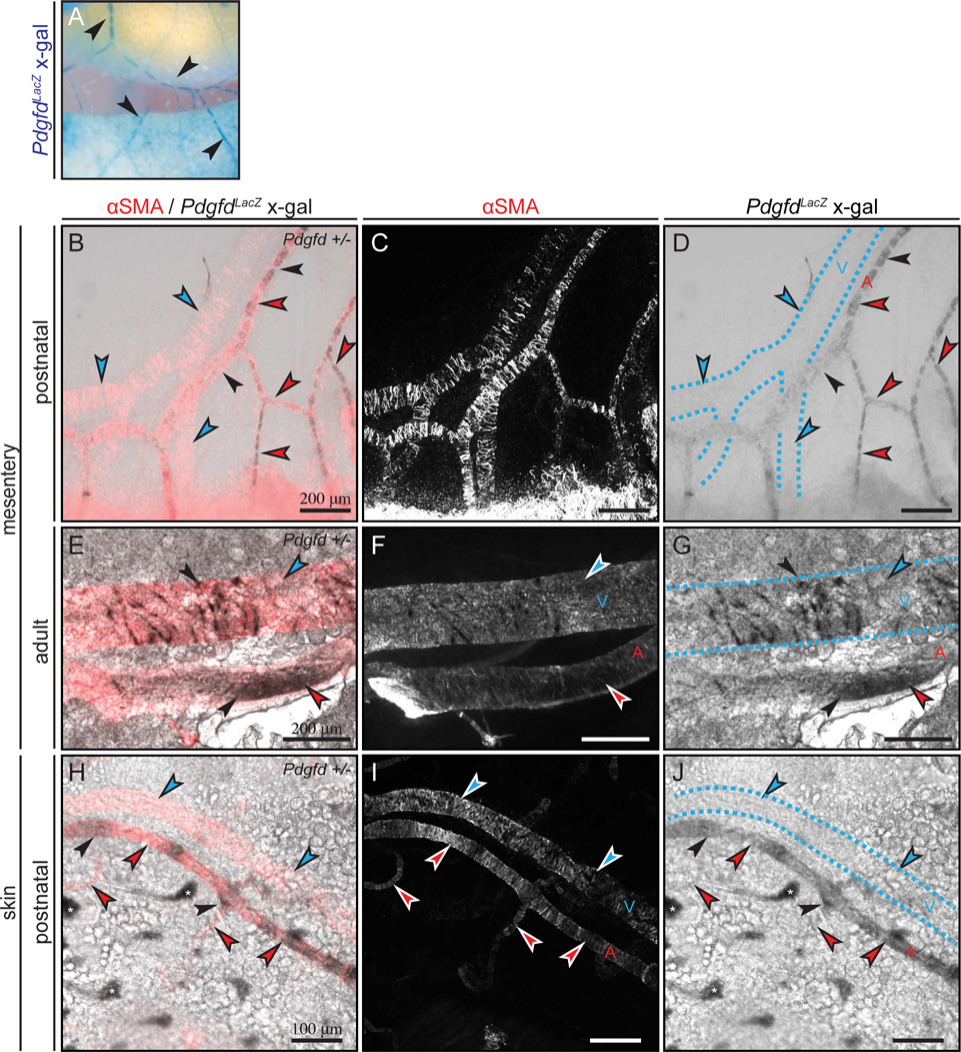
*Pdgfd* is predominantly expressed in arterial endothelial cells, but vSMCs can also express *Pdgfd*. (A) Overview picture showing X-gal stained (black arrows) vessels in whole mount stained postnatal (day P4) mesenteric tissue. (B-J) Whole mount co-stained with X-gal (black arrows) visualized by transmitted light and αSMA to differentiate between arterial (*A*, red arrows) and venous (*V*, blue arrows) vessels. Dashed lines showing veins. (B-C) Representative pictures showing prominent *Pdgfd*^*LacZ*^ expression in postnatal (day P4) mesenteric arteries, but not in veins. (B) αSMA (red) and X-gal (black). (C) αSMA (white), no transmitted light. (D) X-gal (black). (E-G) Representative pictures showing strong *Pdgfd*^*LacZ*^ expression in arteries and distinct *Pdgfd*^*LacZ*^ expressing cells in veins of mesenteric tissue from 16 weeks old mice. (E) αSMA (red) and X-gal (black). (F) αSMA (white), no transmitted light. (G) X-gal (black). (H-J) Representative pictures showing *Pdgfd*^*LacZ*^ expression in arteries of postnatal (day P4) skin tissue, while veins are negative. (H) αSMA (red) and X-gal (black). (I) αSMA (white), no transmitted light. (J) X-gal (black). (*) Indicates hair follicles that appear false positive, because of their structural characteristics. Scale bars 100 μm or 200 μm.

To further explore vascular beds expressing *Pdgfd*, we analyzed postnatal and adult mesenteric tissue for *LacZ* expression. The mesentery has a well-structured vasculature where arteries, veins and lymphatic vessels are clearly distinguishable. Staining of whole mounted postnatal mesenteric tissue revealed a strong vascular *LacZ* expression pattern (Fig 4A). In order to differentiate between arteries and veins, co-staining for *LacZ* expression and αSMA was performed. In postnatal mesentery *LacZ* expression (black arrows) was predominantly found in arteries, characterized by strong, concentric αSMA staining, while veins appeared negative for *LacZ* expression (Fig 4B–4D). Interestingly, as previously observed in the heart (Fig 3A–3C), the *LacZ* expression pattern changed during development into adult tissue. In adult mesentery, *LacZ* was still strongly expressed by arteries. However, distinct *LacZ* expression could be found also in veins (Fig 4E–4G). Furthermore, the *LacZ* expression in adult mesenteric veins was wrapped around the vessels in a typical vSMC-like fashion. The arterial expression was enclosed by the αSMA expressing cells, and thus, likely corresponding to endothelial cells, in both postnatal and adult tissues.

To verify whether the arterial and endothelial expression patterns for *Pdgfd* could be found in other vascular beds, postnatal skin tissue was analyzed, and indeed, a similar endothelial-like arterial *LacZ* expression pattern was found (Fig 4H–4J).

Altogether, the histological analysis of *LacZ* expression in different vascular beds showed that *Pdgfd* is predominantly expressed in arteries, and that both endothelial cells and vSMCs expressed *Pdgfd*, implicating a spatially controlled regulation of *Pdgfd* expression as well as possible region-specific functions of PDGF-D.

### NG2-expressing pericytes are disorganized in the cardiac vasculature of *Pdgfd*^*-/-*^ mice

**Fig 5:**
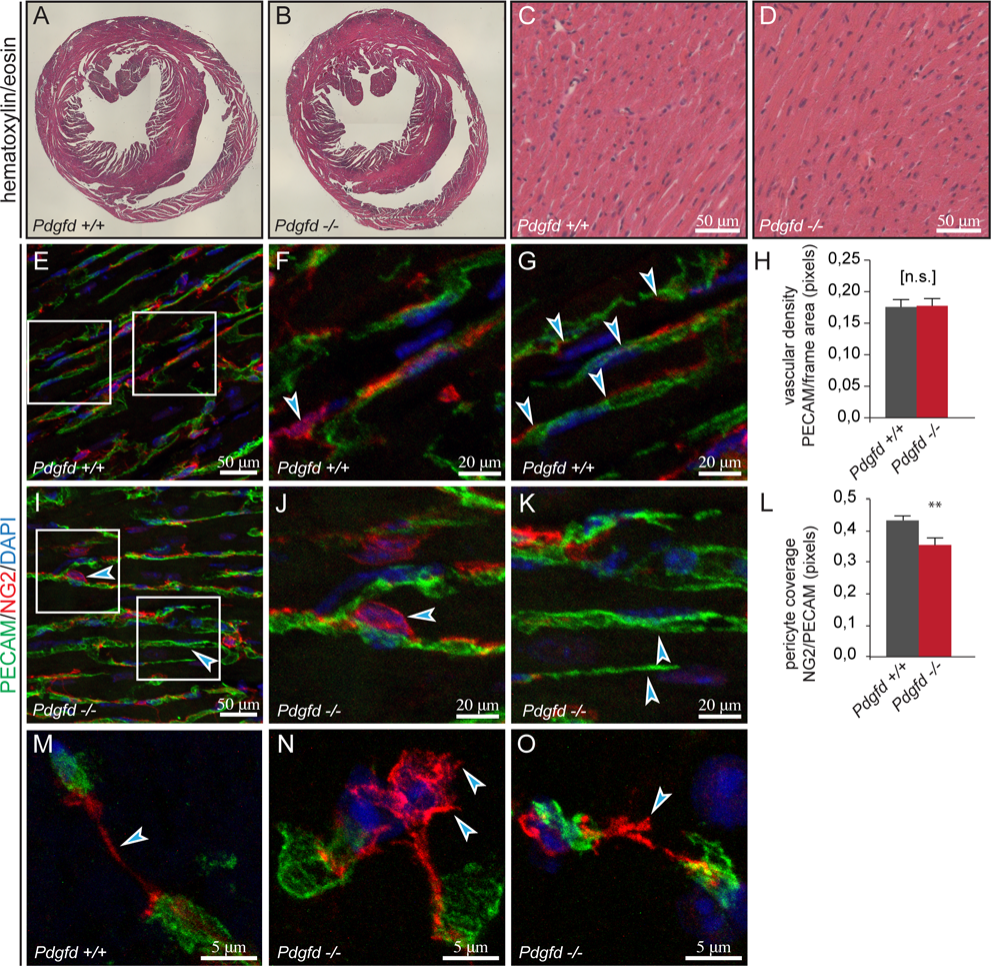
NG2-expressing pericytes are disorganized in the cardiac vasculature of *Pdgfd*^*-/-*^ mice. Representative images from wildtype and *Pdgfd*^-/-^ hearts showing tissue morphology and vascular stainings. (A-D) Hematoxylin and eosin staining showing normal morphology in *Pdgfd*^*-/-*^ hearts compared to *Pdgfd*^+/+^ hearts. (E-G, I-K and M-O) Representative images of PECAM and NG2 staining showing decreased expression of NG2 and altered morphology of NG2-expressing pericytes in *Pdgfd*^*-/-*^ (n = 4) hearts compared with wildtype hearts (n = 4). (E) PECAM and NG2 stained wildtype heart. (F) Magnification of right box in (E), arrow indicating a wildtype pericyte with processes spread out along the vessel. (G) Magnification of the left box in (E), arrows indicating NG2-stained pericyte processes covering wildtype vessels. (H) Quantification of vascular density, PECAM (pixels)/total area. (I) PECAM and NG2 stained *Pdgfd*^*-/-*^ heart. (J) Magnification of the left box in (I), arrow indicating a *Pdgfd*^*-/-*^ pericyte, with a rounder morphology than the corresponding wildtype pericyte in (F). (K) Magnification of the right box in (I), arrows indicating vessel segments not covered by NG2 stained pericyte processes. (L) Quantification of NG2 staining displayed as pericyte coverage, NG2/PECAM pixel ratio. (M-O) Magnifications NG2-positive pericyte processes between vessels, showing abnormal pericyte morphology in *Pdgfd*^*-/-*^. (M) Typical thin wildtype processing. (N) *Pdgfd*^*-/-*^ pericyte with rough body morphology, located between vessels, with thick processes connected to vessels. (O) *Pdgfd*^*-/-*^ pericyte with thick, rough, processes ending abruptly. (A-E) age 12 weeks and (E-O) age 15–17 weeks. Scale bars 20 or 50 μm. Error bars indicating standard deviation. **, p < 0.01.

PDGFRβ signaling is important for proper cardiac development and recruitment of pericytes/vSMCs to blood vessels [10, 11, 26], and previous studies have shown that cardiac overexpression of PDGF-D potently modulates both vascular and connective tissue growth [12]. To investigate potential alterations in the hearts of *Pdgfd-*deficient animals, histological analyses were performed on hearts from age-matched wildtype and *Pdgfd*^-/-^ animals. Hematoxylin and eosin staining showed normal morphology in *Pdgfd*^-/-^ hearts compared to control hearts (Fig 5A–5D). To further evaluate the vascularization in *Pdgfd-*deficient hearts, cryosections were stained with antibodies against PECAM and either PDGFRβ, or NG2, and visualized by immunofluorescence. The *Pdgfd*^-/-^ hearts showed a normal vascular bed with no obvious abnormalities (Fig 5E and 5I). This was confirmed by quantification of vascular density as PECAM pixels per frame (Fig 5H).

To evaluate whether populations of vSMCs/pericytes are missing or defective in *Pdgfd*^-/-^ animals, expression of PDGFRβ, and NG2 were quantified. In heart sections stained for PDGFRβ, no obvious differences in the staining pattern were found between wildtype and *Pdgfd*^-/-^ mice and quantification showed normal expression of PDGFRβ in relation to vessel area (S3 Fig). Interestingly, visualization of NG2 revealed a more disorganized expression pattern in *Pdgfd*^-/-^ compared with wildtype hearts (Fig 5E–5O and S4 Fig). In control animals, NG2 positive pericytes were observed in all blood vessels, with the cell bodies closely bound to the vasculature (Fig 5E and 5F and S4 Fig), and the pericyte processes stretched out along the vessel (Fig 5G). *Pdgfd*^*-/-*^ pericytes displayed a rounder morphology, which was perceived as more detached from the vasculature than the corresponding wildtype pericytes (Fig 5I, upper arrow, 5J and 5N and S4 Fig). Further, *Pdgfd*^-/-^ pericyte processes appeared to be less organized, shorter (Fig 5N arrows, 5O, arrow), and some vessel segments were not covered by NG2 stained pericyte processes (Fig 5I, lower arrow and 5K, arrows). At a higher magnification, NG2 positive pericytes typically displayed thin processes between vessels (Fig 5M), while in *Pdgfd*^*-/-*^ hearts, detached pericytes with rough body morphology and thick processes connecting to the vessels occurred (Fig 5N and 5O).

Image quantification of NG2 staining showed reduced NG2 coverage of the vasculature in *Pdgfd*^-/-^ hearts (Fig 5L).

As PDGF-B deficiency is known to gives rise to detached pericytes and leaky vessels [10, 11], vessel permeability was investigated by intravenous injections of an inert fluorescent tracer (Alexa Fluor® 555 Cadaverine). No increase in leakage was found in *Pdgfd*^-/-^ cardiac vasculature compared to control mice (S5 Fig), suggesting that the decrease in NG2 coverage did not influence the permeability of cardiac vessels.

In summary, the *Pdgfd*^-/-^ hearts showed a normal vascular network, however pericyte/vSMC morphology appeared disorganized and vascular coverage by NG2 expressing cells was significantly lower suggesting that PDGF-D might have an important regulatory function for a selected population of mural cells.

### *Pdgfd*^*-/-*^ mice display mild circulatory defects

**Fig 6:**
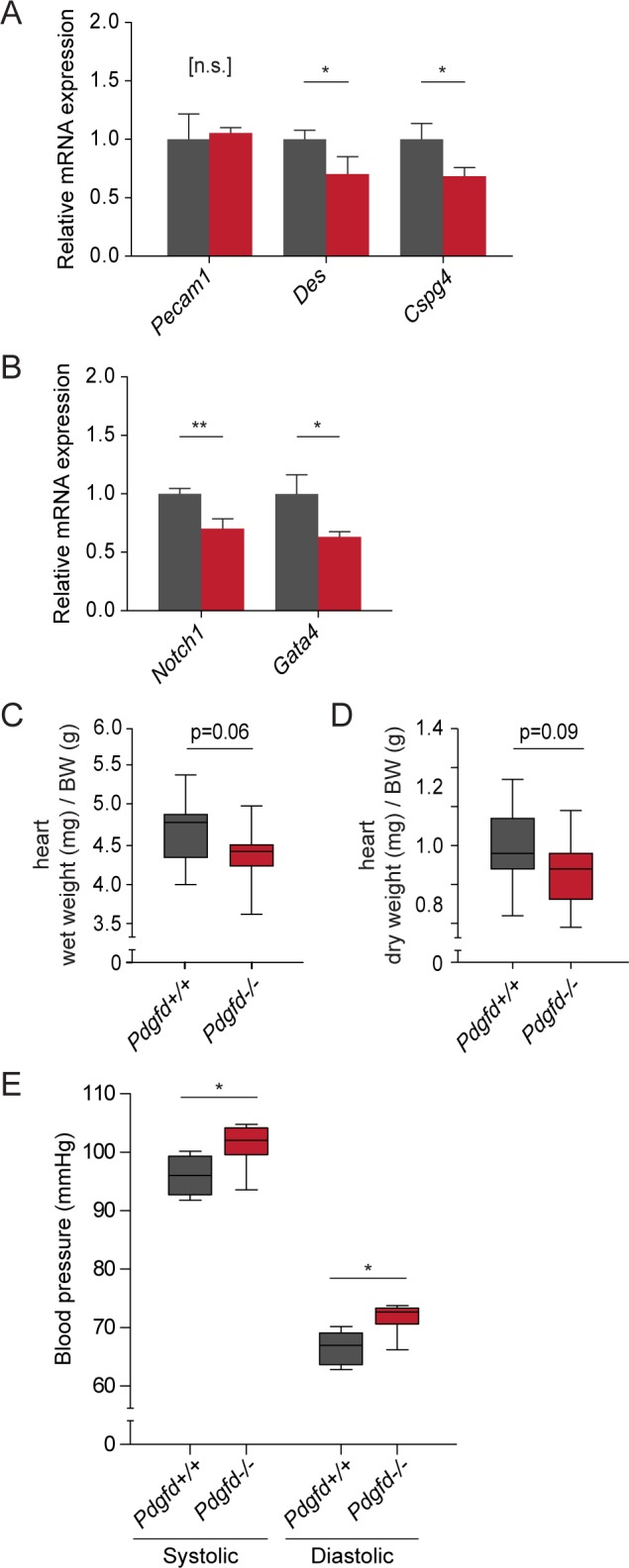
*Pdgfd*^*-/-*^ mice display mild circulatory defects. (A) qPCR analysis on RNA from *Pdgfd*^+/+^ (n = 3) and P*dgfd*^-/-^ (n = 3) mice (aged 18–20 weeks), of endothelial marker *Pecam1*, mural markers *Cspg4* and *Desmin* and (B) developmental markers *Notch1* and *Gata4*. (C-D) Wet and dry weight of hearts from 20–22 weeks old *Pdgfd*^+/+^ (n = 15) and *Pdgfd*^-/-^ (n = 16) mice, normalized to body weight. (E) Tail-cuff blood pressure measurements showing systolic and diastolic blood pressure in *Pdgfd*^+/+^ and *Pdgfd*^-/-^ male mice, aged 17–18 weeks. (Representative experiment, *Pdgfd*^*+/+*^ n = 5 and *Pdgfd*^-/-^ n = 6). In (A-B), error bars indicate standard deviation. In (C-E), boxes indicate 2^nd^ and 3^rd^ quartile, middle bar indicates median and whiskers show min and max, *, p < 0.05 **, p < 0.01.

To investigate whether the disorganized PECAM/NG2-positive vascular network in the cardiac vasculature of *Pdgfd*^*-/-*^ mice has implications on heart function, a gene-expression analysis on age-matched wildtype and *Pdgfd*^*-/-*^ hearts was performed. A broad spectrum of different immunological, developmental and vascular genes was analyzed. Most of the genes tested were not differentially regulated in *Pdgfd*^-/-^ mice. However, *Pdgfd*^-/-^ mice showed reduced mRNA expression of the mural cell markers *Cspg4* (NG2) and *Des* (Desmin) (Fig 6A), confirming the significantly lower levels of NG2 found in the immunofluorescence stainings and supporting the idea that PDGF-D is needed for correct function/differentiation of a certain population of pericytes/vSMCs. The normal appearance of PECAM staining in the histological analysis was also verified by unaltered mRNA expression of the corresponding gene *Pecam1* (Fig 6A). Notably, two additional genes were found to have reduced mRNA expression, namely *Gata4* and *Notch1* (Fig 6B). GATA4 and Notch1 are known to be important for cardiac development and growth, control of cell fate and maintenance of cardiac function in the adult [27–31]. Since the *Pdgfd*^-/-^ animals survive, the role of the reduced expression of *Gata4* and *Notch1* in these mice needs to be further investigated.

To evaluate the cardiac function in *Pdgfd*^-/-^ animals we analyzed heart growth, by measuring heart wet and dry weights (Fig 6C and 6D). The hearts from *Pdgfd*^-/-^ animals appeared to be smaller compared with control animals, although the differences were not statistically significant (wet, p = 0.06 and dry, p = 0.09).

To further analyze possible cardiac defects, systemic blood pressure was measured by non-invasive tail-cuff blood pressure measurements in male *Pdgfd*^-/-^ mice and age-matched male wildtype controls (Fig 6E). *Pdgfd*^*-/-*^ animals displayed a slight but significant elevation in both systolic (+5.2%, *Pdgfd*^+/+^ = 96±3 mmHg, *Pdgfd*^-/-^ = 101±4 mmHg) and diastolic (+10.6%, *Pdgfd*^+/+^ = 66±3 mmHg, *Pdgfd*^-/-^ = 72±3 mmHg) blood pressure compared with the control animals, suggesting a peripheral vascular or cardiac defect in *Pdgfd*^-/-^ mice. To further characterize circulatory deviations in *Pdgfd*^*-/-*^ mice, serum was analyzed for electrolytes (K^+^, Na^+^, Ca^2+^, Cl^-^) and clinical chemistry markers (ALAT, ASAT, ALP and albumin) (Table 2). Most markers were showed no significant difference between wildtype and *Pdgfd*^-/-^ animals, although the male *Pdgfd*^-/-^ mice displayed increased levels of Ca^2+^ and reduced levels of Cl^-^, indicating an imbalance in serum electrolytes.

**Table 2:** Clinical chemistry markers in serum.

	Males				Females			
Serum marker	*Pdgfd*^*+/+*^	*Pdgfd*^*-/-*^	% of *Pdgfd*^*+/+*^	*p-value*^*#*^	*Pdgfd*^*+/+*^	*Pdgfd*^*-/-*^	% of *Pdgfd*^*+/+*^	*p-value*^#^
ALAT (μkat/l)	0.24	0.19	78%	0.24	0.25	0.17	69%	0.21
ASAT (μkat/l)	1.92	1.11	58%	0.08	1.75	1.84	105%	0.85
ALP (mmol/l)	0.93	0.95	101%	0.79	1.65	1.46	88%	0.13
Albumin (g/l)	28.8	29.9	104%	0.23	29.2	28.8	99%	0.69
Calcium, Ca^2+^ (mmol/l)	2.44	2.5	102%	**	2.46	2.46	100%	0.96
Chloride, i-Cl^-^ (mmol/l)	114.6	111.3	97%	*	114.8	114.7	100%	0.99
Potassium, i-K^+^ (mmol/l)	4.23	4.11	97%	0.65	4.03	3.70	92%	0.22
Sodium, i-Na^+^ (mmol/l)	150.4	150.5	100%	0.95	149.9	148.1	99%	0.23

^#^p-value obtained through Student’s t-test,

*, p ≤ 0.05.

**, p ≤ 0.01.

In summary, no major heart defects were found in *Pdgfd*^-/-^ animals, but systemic alterations including slightly elevated blood pressure and altered serum levels of Ca^2+^ and Cl^-^ were observed.

### *Pdgfd*^*-/-*^ mice show normal glucose homeostasis

The observed *Pdgfd*-driven *LacZ* expression around vascular bifurcations suggested that PDGF-D might regulate blood vessel function, and more specifically, regional blood flow, possibly affecting the access of blood borne nutrients.

**Fig 7:**
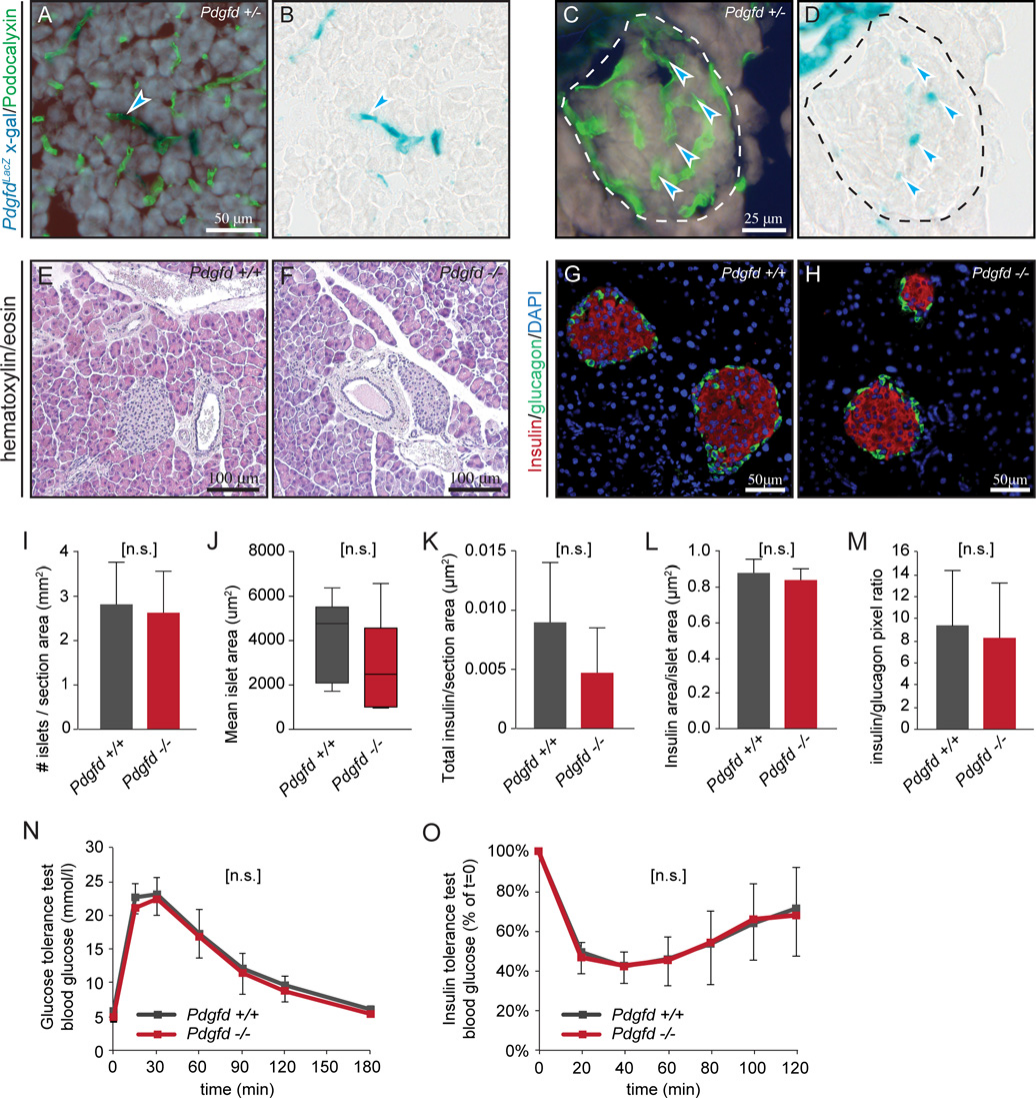
*Pdgfd*^*-/-*^ mice show normal glucose homeostasis. (A-D) Representative images of *Pdgfd*^+/-^ pancreas sections from 21 weeks old mice, showing podocalyxin (green) staining as a marker of endothelial cells and X-gal (blue) staining. (A) Exocrine pancreas, X-gal stained vessel (arrow) and (B) same section without podocalyxin staining. (C) Endocrine pancreas, X-gal staining co-expressed with podocalyxin (arrow) and (D) same section without podocalyxin staining. Dashed lines denotes the pancreatic islet. Representative images from wildtype and *Pdgfd*^-/-^ pancreas showing tissue morphology and different vascular stainings. (E-F) Hematoxylin and eosin staining showing normal morphology in *Pdgfd*^*-/-*^ pancreas compared with wildtype pancreas, from 18 weeks old mice. (G-H) Representative images of wildtype (n = 6) and *Pdgfd*^-/-^ (n = 6) pancreatic islets from 18 weeks old mice, stained for insulin and glucagon. Scale bars, 50μm. (I-M) Quantifications of stainings in (G-H), (I) number of islets per section area, (J) mean islet area, (K) total insulin area per section area, (L) insulin area per islet area, (M) insulin to glucagon ratio. (N-O) Glucose tolerance test in mice aged 17–20 weeks, and insulin tolerance tests in mice aged 19–23 weeks, *Pdgfd*^+/+^ (*n* ≥12) and *Pdgfd*^-/-^ (n≥15) mice. Error bars indicating standard deviation. In (J), boxes indicate 2^nd^ and 3^rd^ quartile, middle bar indicates the median and whiskers show min and max. *, p < 0.05. ***, p < 0.001.

To assess possible metabolic changes in *Pdgfd*^-/-^ mice, the pancreas was analyzed. Similar to heart, both whole mounts and frozen sections from adult *Pdgfd*^+/-^ mouse pancreas displayed a distinct vascular *LacZ* expression pattern (S5 Fig), which was especially strong in medium-sized vessels (Fig 7B and S6 Fig). In endocrine islets, vascular *LacZ* expression was more limited (Fig 7D and S6 Fig). Control stained wildtype whole mounts and sections showed minor background staining (S6 Fig). To define the specific cell type expressing *Pdgfd* in pancreatic blood vessels, X-gal stained frozen sections were immunohistochemically co-stained with PECAM (brown), or podocalyxin (green). Distinct co-localization of endothelial markers and X-gal staining was observed, indicating that endothelial cells express *Pdgfd* in both exocrine (Fig 7A and 7B, arrows and S6 Fig) and endocrine pancreatic vasculature (Fig 7C and 7D and S6 Fig). However, in some blood vessels in the exocrine pancreas, *LacZ* expression appeared to be localized outside the area of endothelial staining (S6 Fig), indicating a non-endothelial expression. The general morphology of *Pdgfd*^*-/-*^ pancreas was investigated by hematoxylin and eosin staining and found to be normal compared to wildtype controls (Fig 7E and 7F). To verify whether the alterations in *Pdgfd*^*-/-*^ *cardiac* blood vessel architecture could also be found in other vascular beds, vascularization and pericyte coverage were investigated in *Pdgfd*^*-/-*^ pancreas. The visualization of blood vessels with podocalyxin and NG2 revealed no significant differences in *Pdgfd*^-/-^ compared to wildtype pancreas, neither in exocrine (S7 Fig) nor in endocrine tissue (S7 Fig). This was verified by quantification of the podocalyxin and NG2 staining (S7 Fig).

To further assess the islet morphology, immunohistochemical stainings for insulin and glucagon, the two major regulators of glucose homeostasis, were performed (Fig 7G and 7H). Quantitative analysis of the stainings showed that the number of islets was unaltered in *Pdgfd*^-/-^ mice as compared to wildtype controls (Fig 7I). Notably, *Pdgfd*^-/-^ mice showed a trend towards smaller islets, although the differences did not reach statistical significance (p = 0.21) due to large standard deviations (Fig 7J). *Pdgfd*^-/-^ mice also showed a trend towards smaller total insulin producing area compared with control mice (p = 0.19), measured as total insulin staining per section area (Fig 7K). However, when measuring individual islets, the area proportion of insulin per islet appeared normal (Fig 7L). *Pdgfd*^-/-^ mice also had a normal ratio of insulin to glucagon staining within the islet compared to control mice (Fig 7M).

To investigate the function of endocrine pancreas in *Pdgfd*^-/-^ mice, a classical glucose tolerance test was performed. *Pdgfd*^-/-^ mice demonstrated a capacity for glucose clearance comparable to wildtype animals (Fig 7N), suggesting that the pancreatic islets have a normal function. To exclude that *Pdgfd*^-/-^ mice have higher insulin sensitivity, they were also subjected to an insulin tolerance test, but showed no alterations in insulin sensitivity compared to controls (Fig 7O). In summary, the *Pdgfd*^-/-^ pancreas showed a normal morphology and vascular network. Although no alterations were seen in the capacity for glucose clearance, or insulin sensitivity, a trend towards smaller islets was observed in *Pdgfd*^-/-^ animals.

## Discussion

The biological function of PDGF-D has so far been mostly characterized from a pathological perspective. Here, we present a *Pdgfd* knockout mouse line (*Pdgfd*^-/-^), carrying a LacZ reporter gene used to visualize *Pdgfd* promoter activity. This model is the first of its kind, where the role of PDGF-D can be studied under physiological conditions, both during development and adulthood. In addition, the *Pdgfd* deficient mice provide a useful, unique tool for mapping of *Pdgfd* expression by visualizing the LacZ reporter gene activity in mouse tissues. Until now, effective tools for such studies have been lacking.

The basic phenotypic studies of *Pdgfd*^*-/-*^ mice showed only minor abnormalities, such as a lower birth rate and slightly reduced survival of first generation offspring from homozygous breedings. These differences may originate from a mild developmental defect in the *Pdgfd*^*-/-*^ embryos, altered maternal capability of the *Pdgfd*^*-/-*^ females to carry out a pregnancy or nurse after delivery. The lack of a well-defined phenotype in C57BL/6 *Pdgfd*^*-/-*^ mice shows that PDGF-D is not crucial for embryonic development or postnatal survival. Thus the *Pdgfd*^*-/-*^ mouse line offers the opportunity to further study the physiological and pathological roles of PDGF-D in the adult mouse.

We investigated *Pdgfd* expression in detail in order to locate relevant sites for biological implications of PDGF-D signaling. Our expression mapping revealed that *Pdgfd* promoter-driven *LacZ* expression was seen in all analyzed tissues. Although the *Pdgfd*-expressing cell types varied between tissues, the vasculature was found to be a common expression site of *Pdgfd*.

Our earlier studies have shown that PDGF-D specifically binds to, and activates, PDGFRβ [3] Interestingly, our present study clearly demonstrates that PDGF-D has a vascular expression pattern, thereby indicating a particular role of PDGF-D in vascular PDGFRβ signaling.

We also found that *Pdgfd* deficiency in mice is compatible with life, in contrast to *Pdgfb* or *Pdgfrb* deficiency. This important, so far missing, piece of information in PDGF biology illustrates that PDGF-B is sufficient for PDGFRβ signaling during embryonic development, and is in line with previous studies showing that mice lacking PDGFRβ, or the ligand PDGF-B, display almost identical, perinatally lethal, phenotypes, characterized by defects in mural cell recruitment [5].

The spatial coexistence of PDGF-D and PDGF-B suggests a specific physiological role of the PDGF-D/PDGFRβ signaling cascade during adult vascular maintenance and homeostasis. We therefore focused our study on adult animals, and a more detailed analysis of *Pdgfd* expression in different vascular beds revealed a distinct arteriolar expression, with an especially strong patterning around vascular bifurcations. Additionally, we observed that mainly endothelial cells, but also certain populations of vSMCs/pericytes, expressed *Pdgfd*, suggesting that PDGF-D may provide both paracrine and autocrine signals to PDGFRβ present on vascular mural cells.

The striking *Pdgfd* expression around vascular bifurcations indicates a region-specific function for PDGF-D at these sites. Since blood flow into capillaries is regulated locally in the arterioles by vSMC contraction and relaxation [32], it is likely that PDGF-D may regulate vSMC/pericyte tone at arteriolar branching points, and thereby modulates local blood flow into capillaries. To explore this hypothesis, we performed blood pressure measurements, and notably, an elevation in blood pressure in *Pdgfd*^-/-^ animals was detected. This might be indicative for an increased vascular resistance in peripheral tissues, which could be caused by perturbations in vSMC actions. However, the changes in systemic blood pressure can also be a result of altered cardiac output, and whether the change in systemic blood pressure observed in *Pdgfd*^*-/-*^ mice is caused by changes in peripheral vascular resistance or cardiac output, or both, needs to be further evaluated.

Furthermore, as vSMCs regulate the blood flow from arterioles to capillaries upon local demand of oxygen, glucose and nutrients of the tissue [32, 33], any severe alterations of capillary blood flow due to lack of PDGF-D should be reflected in the general metabolism. However, as the *Pdgfd*^*-/-*^ mice showed no alterations in glucose clearance from the blood, or in insulin sensitivity, the putative role of PDGF-D is likely more in fine-tuning of the regulation of vSMC/pericyte function.

Analysis of the blood chemistry revealed a modest increase in serum Ca^2+^ concentrations in *Pdgfd*^-/-^ mice. Whether this change in Ca^2+^ levels comes from hormonal changes or originates in altered kidney function, and the role of PDGF-D remains to be elucidated. However, disturbed, Ca^2+^ homeostasis might contribute to altered regulation of vSMC/pericyte function. Ca^2+^ is needed for proper regulation of contraction of vSMCs, cardiomyocytes and other muscle cells [32], and dysfunction within Ca^2+^-dependent pathways may lead to abnormal muscle contraction, which is often seen in cardiovascular diseases [34].

PDGFs biology has been associated with both physiological and pathological regulation of SMCs. For instance, in adult rat, PDGF-B is involved in SMC contraction to regulate interstitial fluid pressure [35], a physiological feature that seems to be controlled also by PDGF-D [18]. Further, PDGF-D is described in several studies to be a potent modulator of the adult vasculature and a mediator of SMC phenotypic modulation [12, 13, 18, 36]. All these data have, however, either been obtained from PDGF-D overexpression studies or from pathological settings. In this study we provide evidence for a vascular function of PDGF-D during physiological conditions where PDGF-D might be involved in normal regulation of vSMC function.

Given that insufficient PDGFRβ signaling is associated with defects in vSMC/pericyte coverage, this was investigated in *Pdgfd*^*-/-*^ mice. In heart tissue we found a defective vascular coverage by mural cells, where vSMCs/pericyte morphology appeared disorganized and not properly attached to the blood vessels, but no vascular leakage was observed. The functional significance of the vSMCs/pericyte changes and whether PDGF-D actually is involved in e.g. regulation of vascular tone need to be further investigated. However, we speculate that this could reflect that only a specific population of vSMCs/pericytes is affected in *Pdgfd*^-/-^ animals, possibly the same population that regulates vascular tone and blood flow to smaller vessels.

Previous studies have shown that lack of PDGFRβ signaling is related to a complex heart phenotype with defects in ventricular septum formation and development of atrioventricular valves, and might be of importance in congenital heart disease [5, 26]. Even though no gross malformation was observed in adult *Pdgfd*^*-/-*^ heart tissue, we found reduced mRNA expression of *Gata4* and *Notch1*, two genes involved in cardiac development, maintenance of adult cardiac function and control of cell fate and differentiation, as well as in congenital heart disease [27–31]. The lower levels of *Gata4* and *Notch1* mRNA were not further investigated, however, this observation suggests a minor disturbance in the maintenance of *Pdgfd*^*-/-*^ heart tissue, which might be connected to the alterations in cardiac mural cell maturation and differentiation.

Notably, in tumor settings, down-regulation of PDGF-D has been shown to decrease expression of Notch-1 [37].

In summary, through detailed characterization of *Pdgfd* expression, followed by a broad vascular analysis and different physiological experiments, we suggest that PDGF-D may have a role in regulation of arterial blood pressure, and we describe a possible role for PDGF-D in mural cell physiology and regulation of vascular homeostasis. The fact that the PDGF family members, including PDGF-D, are implicated in a range of severe diseases augments the importance of fully understanding the diversity and complexity of signaling derived from the PDGF ligand/receptor system. We have created a *Pdgfd-*deficient mouse that will serve as an important platform for further studying the role of PDGF-D and PDGFRβ signaling, providing new insights into the functional relevance of PDGF-D signaling in both physiological and pathological conditions.

## Supporting Information

S1 Fig Weights of wildtype and *Pdgfd*^*-/-*^ mice at an age of 0–16 weeks. Error bars the indicate standard deviation.
(TIF)

S2 Fig Anti-PDGF-D staining of tissues from wildtype and *Pdgfd*^*-/-*^ mice. Representative images of tissues from wildtype and *Pdgfd*^-/-^ mice, stained with an anti-PDGF-D antibody, showing positive staining in wildtype tissues. *Pdgfd*^-/-^ tissues show no, or only limited, staining at the corresponding sites. (A-D) Heart, coronary vessels (arrows). (E-H) Heart, myocardium (arrows). (I, K) Pancreas. (J, L) Pancreas, higher magnification. Endocrine islets (indicated by dashed lines), with vascular staining (arrows) (M, O) Kidney. (N, P) Renal tubuli (arrows), higher magnification. Scale bars 50 μm or 100 μm.
(TIF)

S3 Fig *Pdgfd*^*-/-*^ mice have normal PECAM/PDGFRβ-positive vascular network in the heart. (A-F) Representative images of PECAM and PDGFRβ staining showing normal vascular network in *Pdgfd*^*-/-*^ (n = 3) hearts and normal appearance of PDGFRβ staining compared to wildtype (n = 4) hearts. (A, D) Merged, PECAM (green) and PDGFRβ (magenta) staining. (B, E) PDGFRβ. (C, F) PECAM. (G) Quantification of PDGFRβ staining, PDGFRβ/PECAM pixel ratio. Scale bars 50 μm. Error bars indicating standard deviation.
(TIF)

S4 Fig *Pdgfd*^*-/-*^ mice have disorganized PECAM/NG2-positive vascular network in the heart. The same images of PECAM and NG2 stained heart as shown in Fig 5E and 5I, with PECAM and NG2 channels shown separately, displaying vascular segments deficient of NG2 coverage in *Pdgfd*^*-/-*^ animals.
(TIF)

S5 Fig Fluorescent tracers showing intact vessel integrity in *Pdgfd*^*-/-*^ mice. Representative images from wildtype and *Pdgfd*^-/-^ mice injected with Alexa Fluor 555 Cadaverine, a fluorescent tracer. (A-B) Heart. (C-D) Kidney was used as a positive control for successful injection.
(TIF)

S6 Fig X-gal stainings indicating *Pdgfd* expression in pancreatic endothelium. (A) Adult whole mount pancreas from *Pdgfd*^+/+^ (left) and *Pdgfd*^+/-^ (right) mice showing X-gal staining. (B-D) Representative images of pancreas sections showing X-gal staining (B) *Pdgfd*^+/+^, control. (C) Overview of *Pdgfd*^+/-^ pancreas. (D) Magnification of *Pdgfd*^+/-^ blood vessel and pancreatic duct. (E-H) Representative images of *Pdgfd*^+/-^ pancreas sections showing PECAM (brown) staining as a marker of endothelial cells, X-gal (blue) staining and H&E as counterstaining. (E) Exocrine pancreas. (F) Exocrine pancreas, X-gal staining not expressed with PECAM staining (arrows). (G-H) Pancreatic islets of different sizes, X-gal staining co-localized with PECAM staining (arrows). Dashed lines denotes pancreatic islet. Scale bars 50 μm or 100 μm.
(TIF)

S7 Fig Podocalyxin and NG2 staining showing normal vascular density and NG2 pericyte coverage in *Pdgfd*^*-/-*^ pancreas. (A-C, E-G, I-P) Podocalyxin and NG2 stainings showing normal vascular appearance and normal expression of NG2 in *Pdgfd*^*-/-*^ (n = 3) pancreas, compared with wildtype controls (n = 3). (A-H) Exocrine pancreas. Representative images showing (A, E) merged, podocalyxin (green) and NG2 (red) staining (B, F) NG2 and (C, G) podocalyxin. (D) Quantification of podocalyxin staining displayed as vascular density, podocalyxin pixels/total area. (H) Quantification of NG2 staining showed as NG2/podocalyxin pixel ratio. (I-S) Endocrine pancreas. Representative images showing (I, L) merged, podocalyxin (green) and NG2 (red) staining, (J, Q) NG2 and (K, P) podocalyxin (Q-S) Podocalyxin (green) staining in endocrine pancreas showing normal vascular density in *Pdgfd*^*-/-*^ compared to wildtype animals. (Q-R) Representative images. (S) Quantification of vascular density, (podocalyxin pixels/total area). Scale bars 50 μm. Error bars indicating standard deviation. *Pdgfd*^*+/+*^ n = 3 and *Pdgfd*^-/-^ n = 3.
(TIF)

S1 Table Primer sequences. (PDF)

S2 Table Primary antibodies. (PDF)

S3 Table Genotype distribution from heterozygous crossings of *Pdgfd*^*+/-*^. (PDF)
