# Supplementary figures and images for: Mice Lacking Platelet-Derived Growth Factor D Display a Mild Vascular Phenotype
Source: PLoS One. 2016 Mar 31;11(3):e0152276. doi: 10.1371/journal.pone.0152276 (PMC4816573; doi:10.1371/journal.pone.0152276)

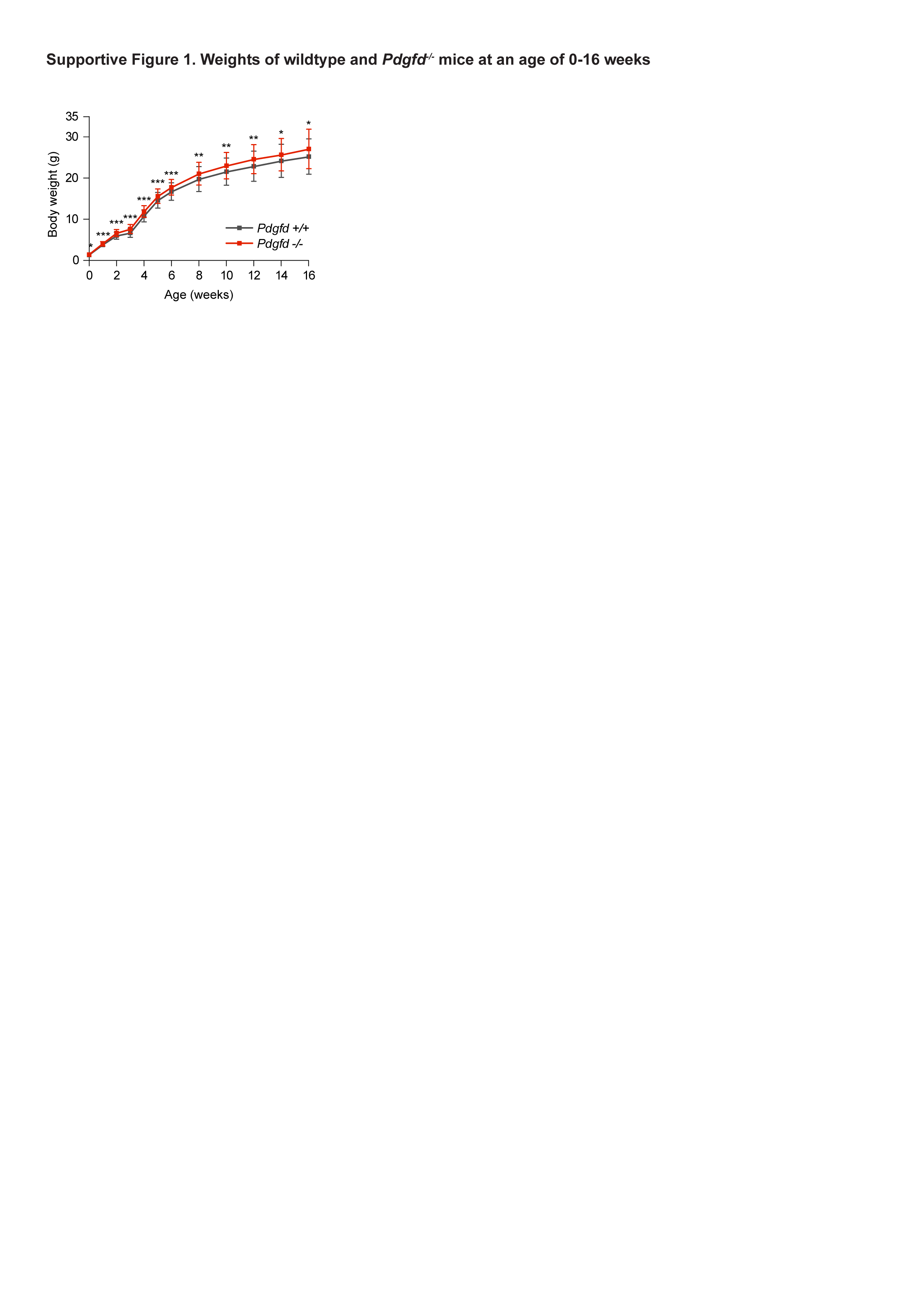

Supplement: S1 Fig — Error bars the indicate standard deviation. (TIF) [file pone.0152276.s001.tif]

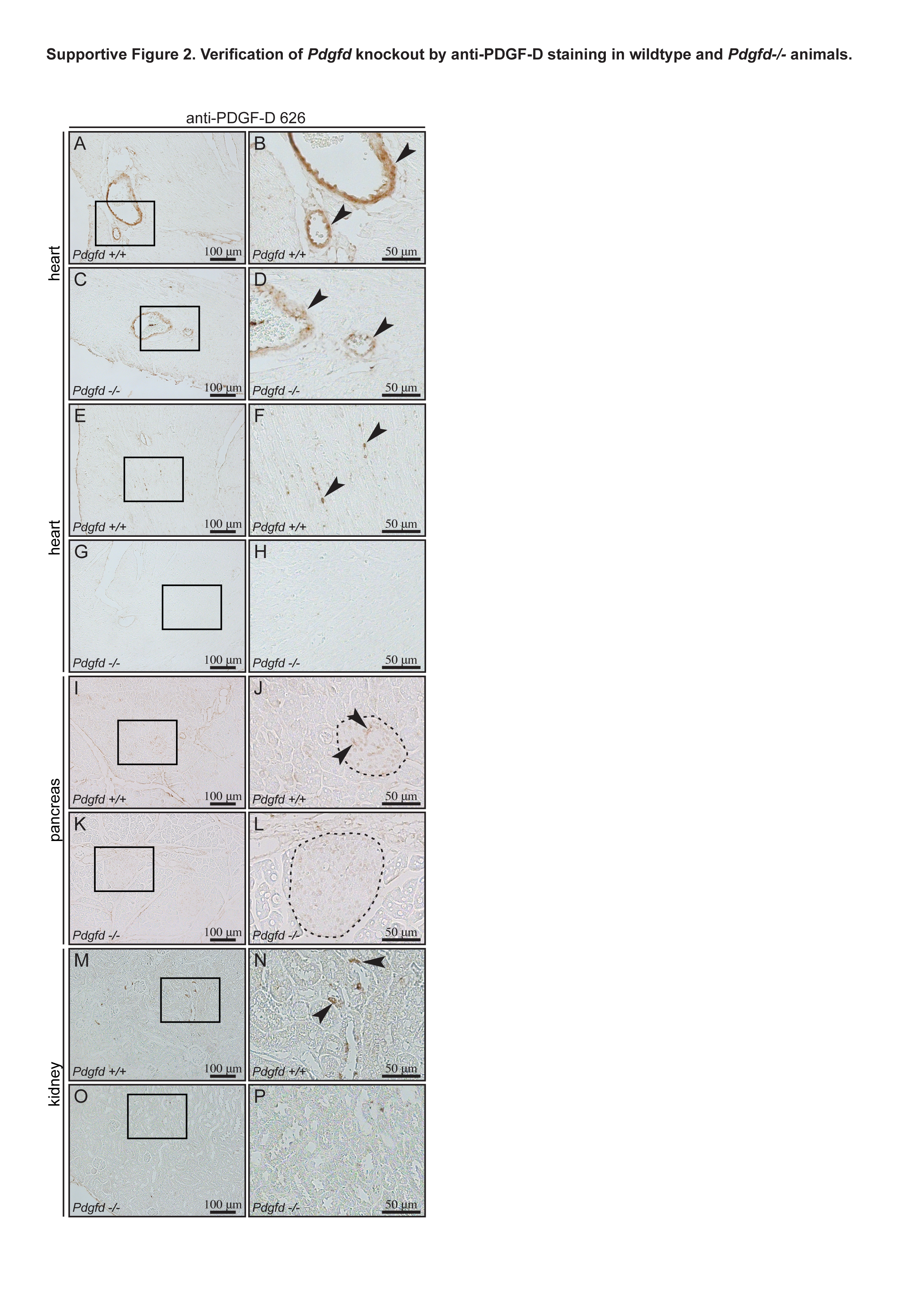

Supplement: S2 Fig — Representative images of tissues from wildtype and Pdgfd-/- mice, stained with an anti-PDGF-D antibody, showing positive staining in wildtype tissues. Pdgfd-/- tissues show no, or only limited, staining at the corresponding sites. (A-D) Heart, coronary vessels (arrows). (E-H) Heart, myocardium (arrows). (I, K) Pancreas. (J, L) Pancreas, higher magnification. Endocrine islets (indicated by dashed lines), with vascular staining (arrows) (M, O) Kidney. (N, P) Renal tubuli (arrows), higher magnification. Scale bars 50 μm or 100 μm. (TIF) [file pone.0152276.s002.tif]

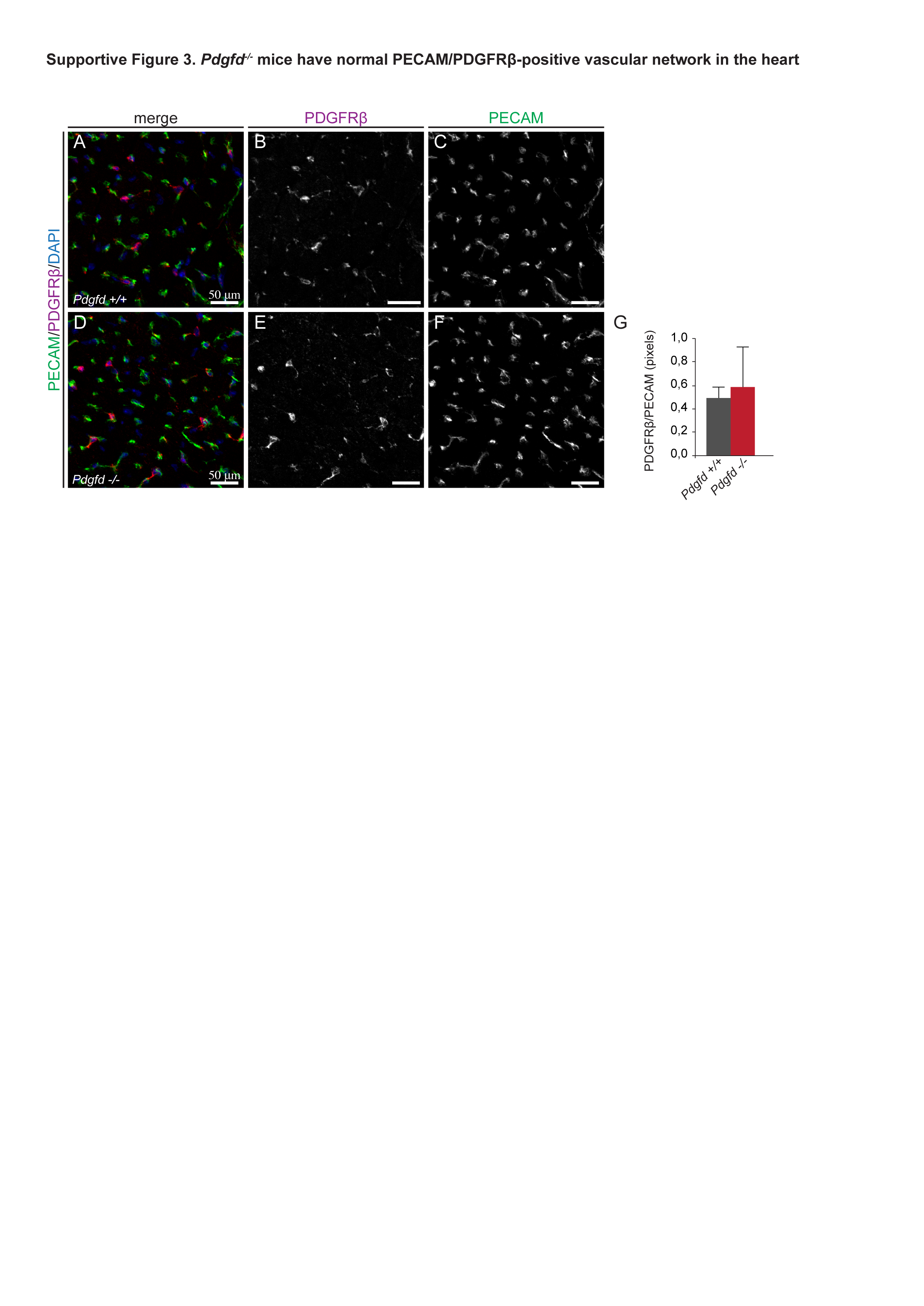

Supplement: S3 Fig — (A-F) Representative images of PECAM and PDGFRβ staining showing normal vascular network in Pdgfd-/- (n = 3) hearts and normal appearance of PDGFRβ staining compared to wildtype (n = 4) hearts. (A, D) Merged, PECAM (green) and PDGFRβ (magenta) staining. (B, E) PDGFRβ. (C, F) PECAM. (G) Quantification of PDGFRβ staining, PDGFRβ/PECAM pixel ratio. Scale bars 50 μm. Error bars indicating standard deviation. (TIF) [file pone.0152276.s003.tif]

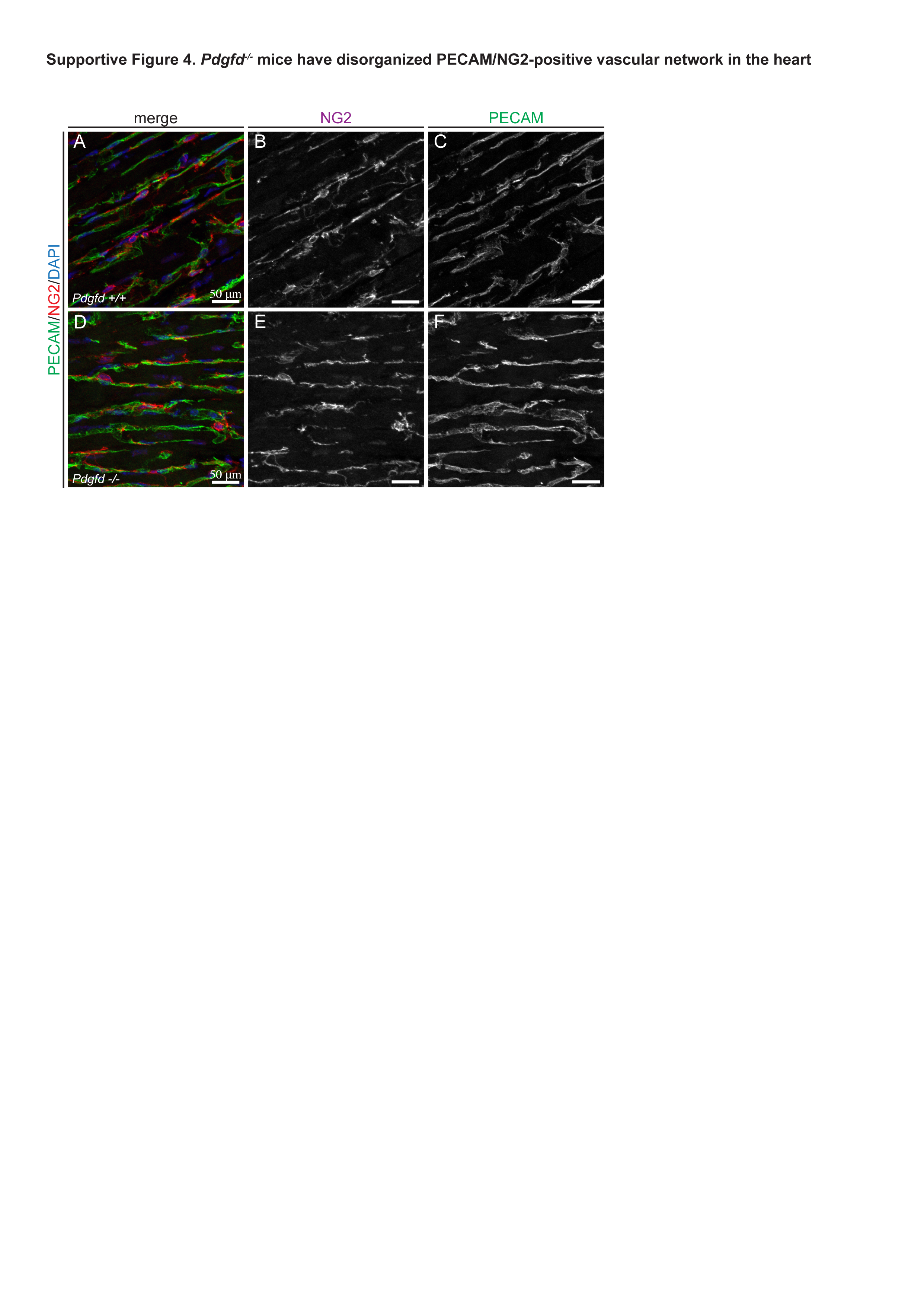

Supplement: S4 Fig — The same images of PECAM and NG2 stained heart as shown in Fig 5E and 5I, with PECAM and NG2 channels shown separately, displaying vascular segments deficient of NG2 coverage in Pdgfd-/- animals. (TIF) [file pone.0152276.s004.tif]

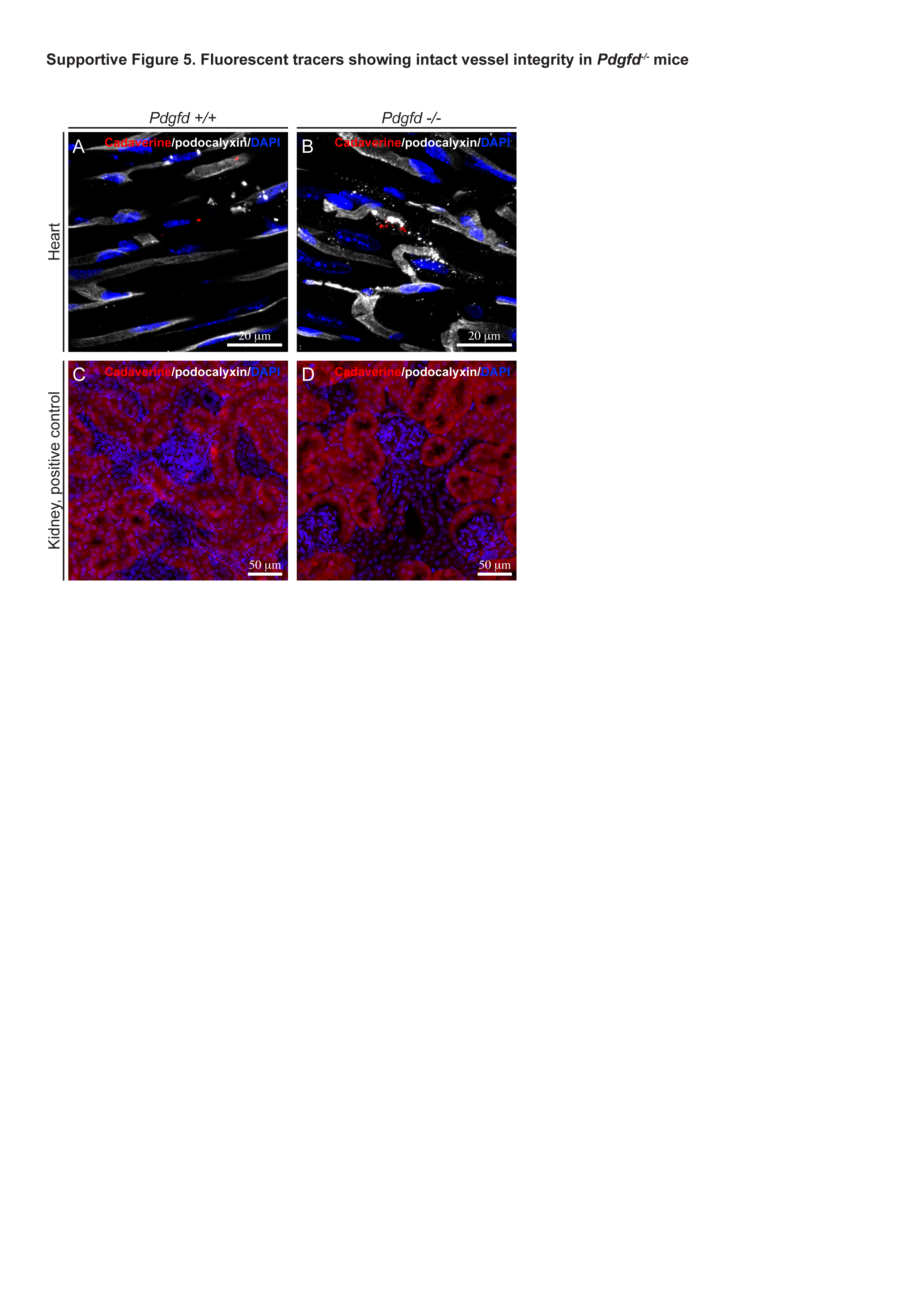

Supplement: S5 Fig — Representative images from wildtype and Pdgfd-/- mice injected with Alexa Fluor 555 Cadaverine, a fluorescent tracer. (A-B) Heart. (C-D) Kidney was used as a positive control for successful injection. (TIF) [file pone.0152276.s005.tif]

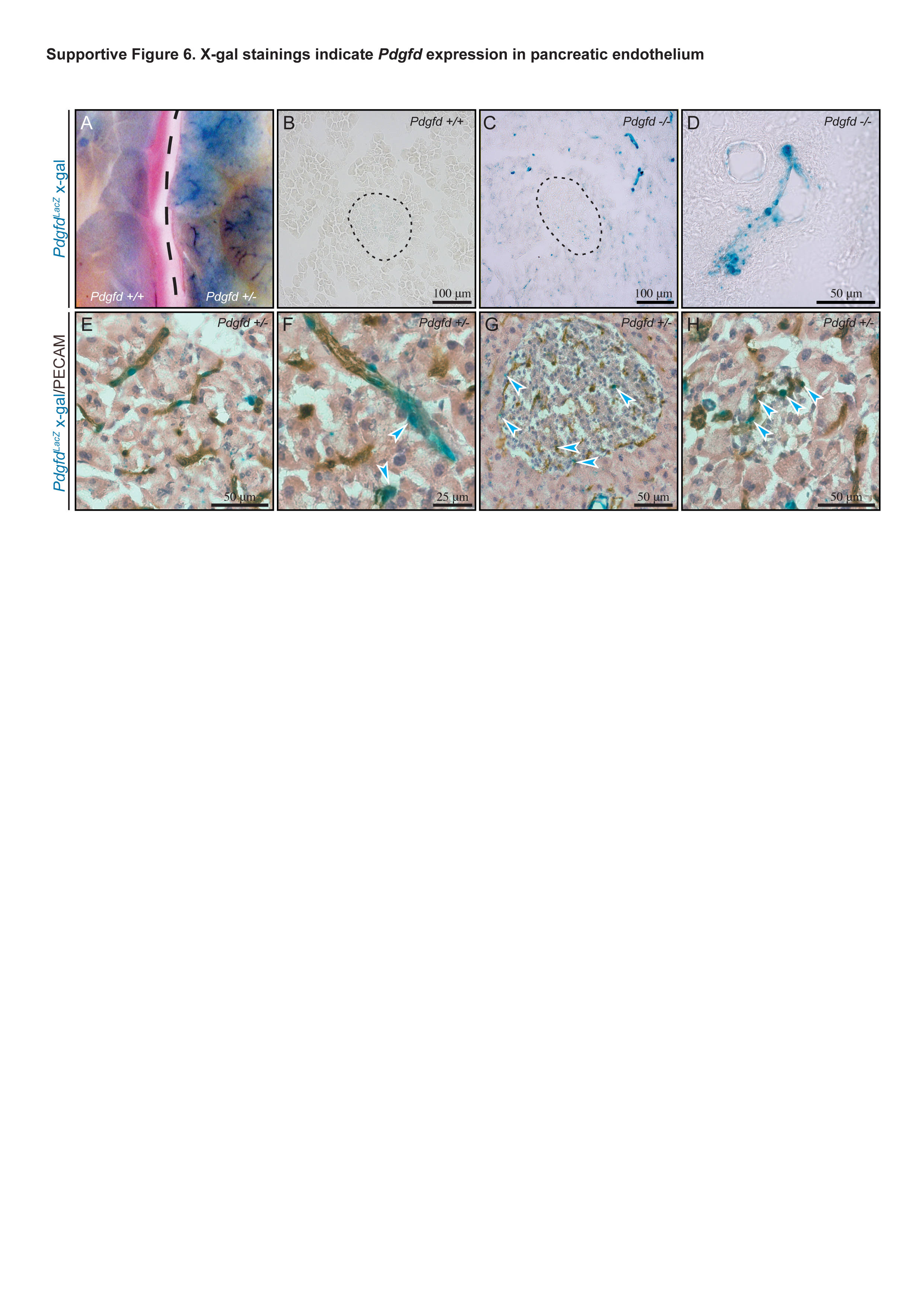

Supplement: S6 Fig — (A) Adult whole mount pancreas from Pdgfd+/+ (left) and Pdgfd+/- (right) mice showing X-gal staining. (B-D) Representative images of pancreas sections showing X-gal staining (B) Pdgfd+/+, control. (C) Overview of Pdgfd+/- pancreas. (D) Magnification of Pdgfd+/- blood vessel and pancreatic duct. (E-H) Representative images of Pdgfd+/- pancreas sections showing PECAM (brown) staining as a marker of endothelial cells, X-gal (blue) staining and H&E as counterstaining. (E) Exocrine pancreas. (F) Exocrine pancreas, X-gal staining not expressed with PECAM staining (arrows). (G-H) Pancreatic islets of different sizes, X-gal staining co-localized with PECAM staining (arrows). Dashed lines denotes pancreatic islet. Scale bars 50 μm or 100 μm. (TIF) [file pone.0152276.s006.tif]

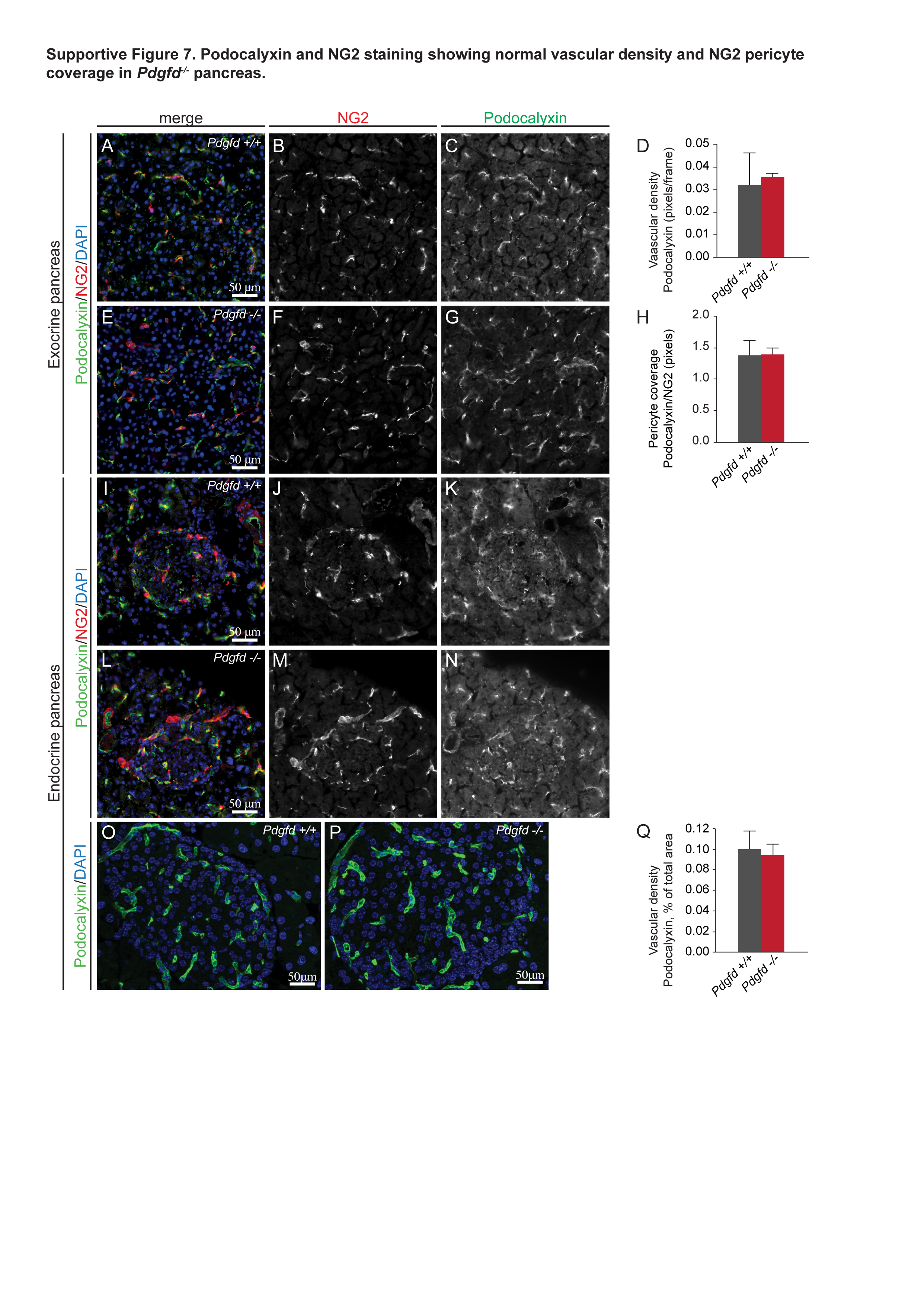

Supplement: S7 Fig — (A-C, E-G, I-P) Podocalyxin and NG2 stainings showing normal vascular appearance and normal expression of NG2 in Pdgfd-/- (n = 3) pancreas, compared with wildtype controls (n = 3). (A-H) Exocrine pancreas. Representative images showing (A, E) merged, podocalyxin (green) and NG2 (red) staining (B, F) NG2 and (C, G) podocalyxin. (D) Quantification of podocalyxin staining displayed as vascular density, podocalyxin pixels/total area. (H) Quantification of NG2 staining showed as NG2/podocalyxin pixel ratio. (I-S) Endocrine pancreas. Representative images showing (I, L) merged, podocalyxin (green) and NG2 (red) staining, (J, Q) NG2 and (K, P) podocalyxin (Q-S) Podocalyxin (green) staining in endocrine pancreas showing normal vascular density in Pdgfd-/- compared to wildtype animals. (Q-R) Representative images. (S) Quantification of vascular density, (podocalyxin pixels/total area). Scale bars 50 μm. Error bars indicating standard deviation. Pdgfd+/+ n = 3 and Pdgfd-/- n = 3. (TIF) [file pone.0152276.s007.tif]
