# Supplementary material for: Mice Lacking Platelet-Derived Growth Factor D Display a Mild Vascular Phenotype
Source: PLoS One. 2016 Mar 31;11(3):e0152276. doi: 10.1371/journal.pone.0152276 (PMC4816573; doi:10.1371/journal.pone.0152276)
Supplement: S1 Table — (PDF) [file pone.0152276.s008.pdf]

Supportive Table 1. Primer sequences

Gene (PCR genotyping)	Forward 5 '- 3'	Reverse 5' - 3'
Pdgfd wildtype exon1 (PCR genotyping)	GAATCCACGTCAACCTGTTG	CGCACAGGAGAATGGAGACT
Pdgfd knockout allele (PCR genotyping)	GAATCCACGTCAACCTGTTG	GTCTGTCCTAGCTTCCTCACTG

Gene (qPCR)	Forward 5 '- 3'	Reverse 5' - 3'
Pdgfd exon 1	GACTCAACCCTTTGGGCTTT	AGCTTTGATGGATGCTCTCTG
Cspg4	GGCTCTTACCTTGGCCTTGT	GGGATGTGGAGAACTGGAGC
Des	GATGGCCTTGGATGTGGAGA	TCAGAACCCCTTTGCTCGG
Gata4	TTCTGGGAAACTGGAGCTGG	TTTCTGCCTGCTACACACCC
Notch1	TTGAGATGCTCCCAGCCAAG	GCTGAGGCAAGGATTGGAGT
Pdgfd	CTTCTGACATGGTGGCTCCG	CTTGGAGGGATCTCCTTGTGG
L19	GGTGACCTGGATGAGAAGGA	TTCAGCTTGTGGATGTGCTC

Gene (Qiagen prevalidated, qPCR)	Qiagen primer name	Catalogue number
Pecam1	Mm_Pecam1_1_SG	QT01052044 (Qiagen)
L19	Mm_Rpl19_2_SG	QT01779218 (Qiagen)
