# Supplementary material for: Mice Lacking Platelet-Derived Growth Factor D Display a Mild Vascular Phenotype
Source: PLoS One. 2016 Mar 31;11(3):e0152276. doi: 10.1371/journal.pone.0152276 (PMC4816573; doi:10.1371/journal.pone.0152276)
Supplement: S2 Table — (PDF) [file pone.0152276.s009.pdf]

Supportive Table 2. Primary antibodies

Antigen	Product identity	Host species	Manufacturer	Dilution
PECAM[1] (mouse)	CD31 AF3628	goat	R&D Systems	1:500
PECAM[2] (mouse)	Platelet Endothelial Cell Adhesion Molecule-1 MEC13.3	rat	BD Pharmingen	1:500
Podocalyxin (mouse)	Podocalyxin AF1556	goat	R&D Systems	1:200
aSMA	Actin, a-Smooth Muscle-Cy3 clone 1A4, C6198	mouse	Sigma-Aldrich	1:2000
PDGFRβ (human)	PDGFRβ (28E1) #3169	rabbit	Cell signaling	1:50
NG2 (rat)	NG2 Chondroitin Sulfate Proteoglycan AB5320	rabbit	Millipore	1:200
Insulin (human)	Insulin MAB1417	rat	R&D Systems	1:600
Glucagon	Glucagon AB932	rabbit	Millipore	1:500
PDGF-D (human)	Anti-PDGF-D 626	rabbit	(in-house-made)	1:500

[1]Used for Fig. 5, S3, S4
[2]Used for Fig. S6
