# Supplementary material for: Mice Lacking Platelet-Derived Growth Factor D Display a Mild Vascular Phenotype
Source: PLoS One. 2016 Mar 31;11(3):e0152276. doi: 10.1371/journal.pone.0152276 (PMC4816573; doi:10.1371/journal.pone.0152276)
Supplement: S3 Table — (PDF) [file pone.0152276.s010.pdf]

Supportive Table 3. Genotype distribution from heterozygous crossings of *Pdgfd*$^{+/-}$ mice

***Pdgfd*$^{+/-}$ x *Pdgfd*$^{+/-}$**

+/+	+/-	-/-	Chi-square
93 (27%)	179 (51%)	78 (22%)	0.48

Number of animals per genotype (percentage of total)
n=350
